# Hybridization in the Subtribe Alopecurinae Dumort. (Poaceae) According to Molecular Phylogenetic Analysis: Different Ploidy Level Tells Different Origin of the Groups

**DOI:** 10.3390/plants13070919

**Published:** 2024-03-22

**Authors:** Alexander A. Gnutikov, Nikolai N. Nosov, Elizaveta O. Punina, Igor G. Loskutov, Victoria S. Shneyer, Sergei A. Chekrygin, Alexander V. Rodionov

**Affiliations:** 1 Department of Genetic Resources of Oat, Barley, Rye, Federal Research Center N. I. Vavilov All-Russian Institute of Plant Genetic Resources (VIR), 190000 St. Petersburg, Russia; a.gnutikov@vir.nw.ru (A.A.G.); i.loskutov@vir.nw.ru (I.G.L.); 2 Laboratory of Biosystematics and Cytology, Komarov Botanical Institute of the Russian Academy of Sciences, 197022 St. Petersburg, Russia; epunina@binran.ru (E.O.P.); shneyer@binran.ru (V.S.S.);; 3 “Center Bio-Bank”, St. Petersburg State University (SPbSU), 199034 St. Petersburg, Russia

**Keywords:** grasses, hybridization, ITS, molecular phylogeny, NGS, Poeae, *rbc*L, *mat*K, *ndh*F

## Abstract

We performed next-generation sequencing of the 18S rDNA–ITS1–5.8S rDNA region along with traditional Sanger sequencing of *rbc*L, *mat*K, *ndh*F, and ITS1–5.8S rDNA–ITS2 to clarify the hybridization pattern in the subtribe Alopecurinae and in the genus *Alopecurus* in particular. Our data support the hybrid origin of *Alopecurus* × *brachystylus* from hybridization between *A. geniculatus* (sect. *Alopecurium*) and *A. pratensis* (sect. *Alopecurus*). Moreover, in the rDNA of hybrid *A.* × *brachystylus*, only *A. aequalis*-like ribotypes from tetraploid *A. geniculatus* participated. Surprisingly, we found the traces of introgression of *A. arundinaceus*-like ribotypes not only in hybrid *A.* × *marssonii* (*A. geniculatus* × *A. arundinaceus*) but in *A. aequalis* s. str. as well. A high-polyploid group from the section *Alopecurus*, *A*. aggr. *alpinus* has undoubted hybrid origin: e. g., *A. brachystachyus* has rDNA from the sect. *Alopecurium*. *Alopecurus alpinus*, with its allies, is clearly distinct from other members of the sect. *Alopecurus* (especially by maternal line) and thus we can re-establish a previous opinion about the separate group to which *A. alpinus* belongs. Species from the section *Colobachne* (presumably Alpine grasses from Ancient Mediterranean region) probably hybridized with the *A. alpinus* group. Even *A. myosuroides* (sect. *Pseudophalaris*) that could be referred to the separate genus has ribotypes common with the species of the section *Alopecurium* (*A. aequalis*, *A. geniculatus*) in one of the accessions. Additionally, we found that the possible polyphyletic origin of the genus *Limnas*. *Limnas stelleri* is very close to *Alopecurus magellanicus* according to NGS data, while *L. malyschevii* is more or less distinct from other studied species of the genus *Alopecurus*.

## 1. Introduction

The subtribe Alopecurinae belongs to the largest tribe of the grass family, Poeae, and is widespread in the temperate zone of both Hemispheres. It comprises the genera *Alopecurus* L. and *Limnas* Trin. according to the most recent data and is closely related to the genera *Beckmannia* Host and *Rhizocephalus* Boiss. [1]. The subtribe Alopecurinae is characterized by very dense, spike-shaped panicles of regular cylindrical form [2]. *Alopecurus*, the main genus of the subtribe, comprises up to 70 species [2]. It is known for drastic differences in the chromosome numbers of the species: from 2n = 14 in *Alopecurus aequalis* Sobol. to 2n = ca. 100 in *A. alpinus* Vill. [3]. This polyploid range most probably originated via interspecific hybridization, which is rather common in the Poeae tribe [1,4,5]. Hybrids in the genus *Alopecurus* have been noted for a long time, and nothospecies have been described beginning from *A.* × *brachystylus* Peterm. (*A. geniculatus* L. × *A. pratensis* L.).

Recent phylogenetic research of the subtribe Alopecurinae confirmed its separation from the subtribe Phleinae Dumort. that has similar inflorescences and the affinity of Alopecurinae with some genera from other subtribes, in particular *Arctophila* (Rupr.) Andersson, *Dupontia* R.Br., *Arctagrostis* Griseb., and, only according to the nuclear gene sequence data, *Arctopoa* (Griseb.) Prob. [1,5,6]. Thus, inflorescence traits of the Alopecurinae members can be explained by parallel evolution when similar structures form independently in two lines of taxa originated from the common ancestor. Additionally, the similar inflorescence has the genus *Lagurus* L. [2] which belongs to wholly different group in the Poeae tribe—Aveninae J.Presl (Aveneae chloroplast group) [1].

Recently, the genus *Alopecurus* is divided into four sections: *Alopecurus*, *Colobachne* (P. Beauv.) Griseb., *Alopecurium* Dumort., and *Pseudophalaris* Tzvelev [2,7]. The section *Alopecurus* differs from other species by their cylindrical to ellipsoidal panicles and absence of palea [2]. This section also can be divided into two groups: tetra- and hexaploid species of *A*. aggr. *pratensis* and *A. arundinaceus* Poir., and high polyploid Siberian and Arcto-Alpine species *A. brachystachyus* M.Bieb., *A. alpinus*, *A. borealis* Trin., *A. glaucus* Less. with related taxa. The section *Colobachne* contains mostly Alpine grasses with short, wide-ellipsoidal panicles and mostly present palea. Members of the sect. *Alopecurium* have an annual or shortly lived perennial habit with geniculate stems and the sect. *Pseudophalaris* is distinguished by annual habit and glumes with winged keel [2].

Molecular phylogenetic analysis revealed some lines within *Alopecurus* that did not correspond with taxonomic division of the genus [1,6] and pointed at the intersectional hybridization events. Previous research of *Alopecurus* mainly covered American and Middle Eastern species [1,6,8,9]. Meanwhile, Asian mountains, for example, Altai Mountain Country as well as Caucasus could provide us many hybrid taxa that have not been analyzed until now (see [10] about the plant speciation centers). New methods of next-generation sequencing (NGS) allow the detection of multiple hybridization events in plants even when their morphology is uniform. Our goal of this work is to trace the hybridization events in the subtribe Alopecurinae (especially in the genus *Alopecurus*, taking into account hybrid mountain species from Altai-Sayan region) that could shed light on phylogenetic relationships in the subtribe, and possibly its relationships with other taxa of the tribe Poeae. For our analysis, we used the sequences of the region 18S rDNA–ITS1–5.8S rDNA obtained by NGS and the sequences of ITS1–5.8S rDNA–ITS2, *mat*K, *rbc*L, and *ndh*F obtained via standard Sanger sequencing.

## 2. Results

Since hybridization is known to occur frequently in the tribe Poeae, we treated chloroplast and ITS datasets separately. In order to trace possible hybridization events and assess geographical variability, we analyzed multiple samples of the same species for some chloroplast datasets.

**Figure 1:**
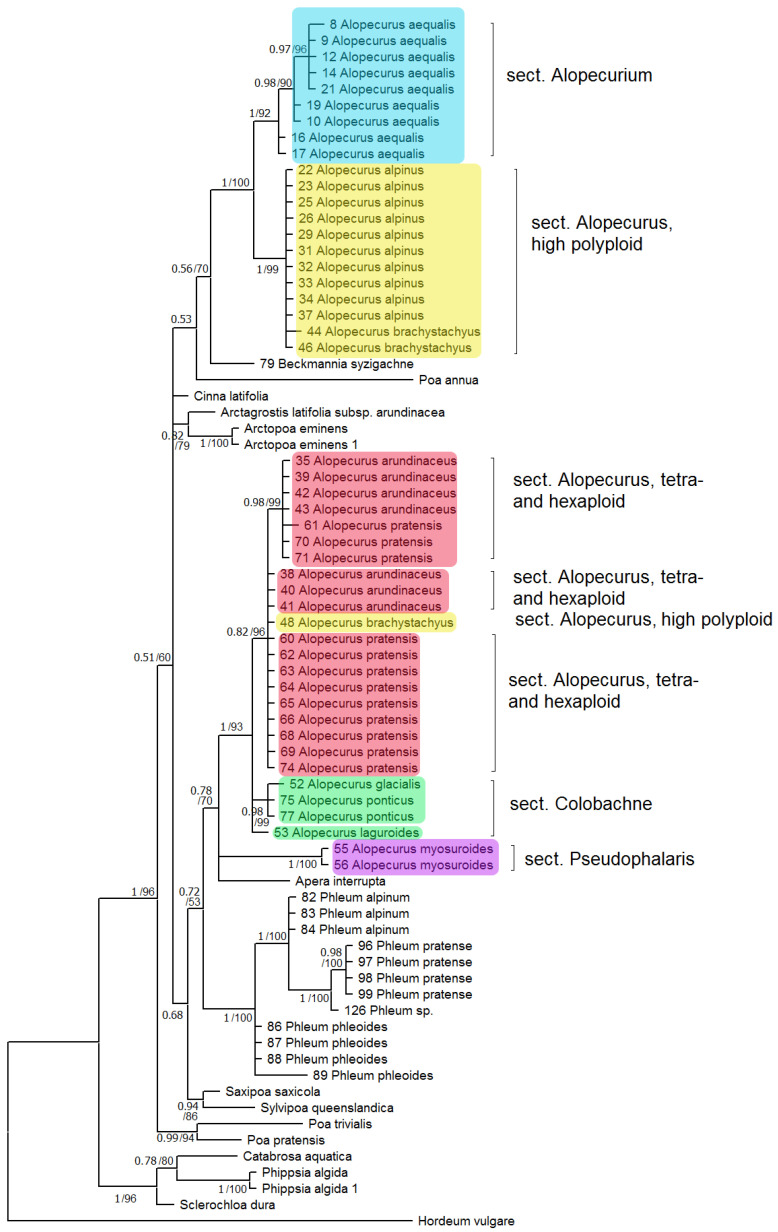
Phylogenetic tree of the subtribe Alopecurinae and related species according to the *rbc*L + *mat*K sequence data. The first index on the branch is the posterior probability in Bayesian inference, the second is the bootstrap index obtained by Maximum Likelihood algorithm. When only one index is shown on the branch it is the posterior probability.

**Figure 2:**
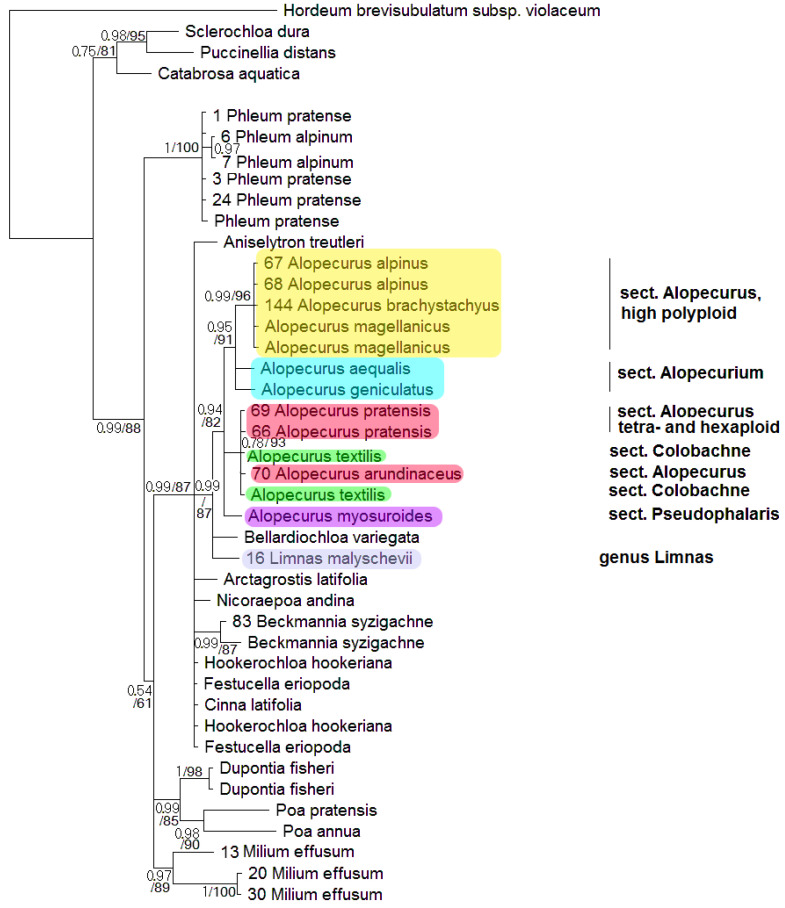
Phylogenetic tree of the subtribe Alopecurinae and related species according to the *ndh*F sequence data. The first index on the branch is the posterior probability in Bayesian inference; the second is the bootstrap index obtained by Maximum Likelihood algorithm. When only one index is shown on the branch, it is the posterior probability.

Phylogenetic tree built on concatenated *rbc*L + *mat*K dataset shows two well-supported clades where the studied samples of the genus *Alopecurus* fall (Figure 1). The first clade (PP = 1, BS = 100) includes section *Alopecurium* (*A. aequalis*) and high polyploid members of the section *Alopecurus* (*A. alpinus*, *A. brachystachyus*). This clade is, in turn, sister to *Beckmannia syzigachne* with low resolution (PP = 0.56, BS = 70). The second clade (PP = 1, BS = 93) contains tetra- and exaploidy species from the sections *Alopecurus* (*A. pratensis* + *A. arundinaceus*), *Colobachne* (*A. glacialis* K.Koch, *A. ponticus* K.Koch), and one sample of *A. brachystachyus* from Irkutsk Oblast, Eastern Siberia, Russia. *A. laguroides* Balansa has an uncertain position in this large clade. *Alopecurus myosuroides* (sect. *Pseudophalaris*) falls into a separate clade (PP = 1, BS = 100) that forms a polytomy with the previously discussed clade of *Alopecurus*, and *Apera* Adans. (PP = 0.78, BS = 70). The second clade, in addition, has weakly supported grouping with the subtribe Phleinae (PP = 0.72, BS = 53). The overall placement of the Alopecurinae species on the tree built by *mat*K sequence data separately is almost the same (Supplementary Figure S2). But in this case intersectional hybrid *A.* × *brachystylus* (*A. geniculatus* × *A. pratensis*) falls within the clade including section *Alopecurium* (we have no *rbc*L data for *A.* × *brachystylus* and thus did not include it in the analysis of concatenated sequences). *Limnas stelleri* Trin. has an uncertain position on the separate *rbc*L tree (Supplementary Figure S1) but we do not have *mat*K sequences of *L. stelleri* for a combined data tree. In phylogenetic analysis based on *ndh*F sequences, we used fewer samples (Figure 2). The Alopecurinae clade groups with *Bellardiochloa variegata* (Lam.) Kerguélen (PP = 0.99, BS = 87), whereas *Beckmannia syzigachne* branches separately (PP = 0.99, BS = 87) within the large clade containing the genus *Alopecurus*, *Hookerochloa hookeriana* (F.Muell. ex Hook.f.) E.B.Alexeev, *Festucella eriopoda* (Vickery) E.B.Alexeev, and *Cinna latifolia* (Trevir. ex Göpp.) Griseb. (PP = 0.99, BS = 87) (Figure 2). The *Alopecurus* clade (PP = 0.94, BS = 82) divides into sect. *Alopecurus* (*A. alpinus + A. brachystachyus + A. magellanicus* Lam.) + sect. *Alopecurium* subclade (PP = 0.95, BS = 91) and a subclade that contains sect. *Alopecurus* (*A. pratensis* + *A. arundinaceus*) + sect. *Colobachne* (*A. textilis* Boiss.) (PP = 0.78, BS = 93) while *A. myosuroides* has a sister position to all other species.

**Figure 3:**
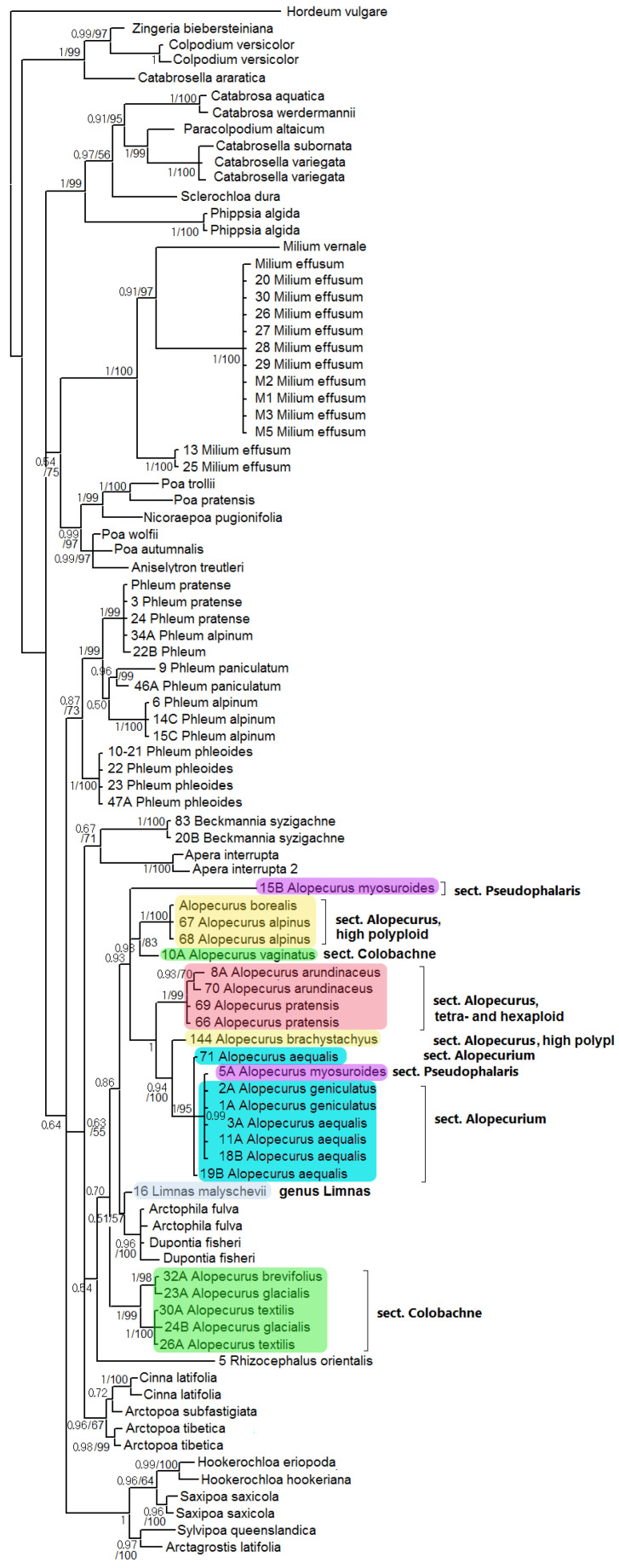
Phylogenetic tree of the subtribe Alopecurinae and related species according to the ITS sequence data. The first index on the branch is the posterior probability in Bayesian inference; the second is the bootstrap index obtained by Maximum Likelihood algorithm. When only one index is shown on the branch, it is the posterior probability.

ITS-inferred phylogeny presents a weakly supported clade of *Beckmannia* + *Apera* (PP = 0.67, BS = 71), subtribe Alopecurinae, and *Cinna* L. + *Arctopoa* (Griseb.) Prob. (PP = 0.63, BS = 55) (Figure 3). This clade forms polytomy with monophyletic Phleinae and *Hookerochloa* E.B.Alexeev + *Saxipoa* Soreng, L.J.Gillespie & S.W.L.Jacobs + *Sylvipoa* Soreng, and L.J.Gillespie & S.W.L.Jacobs + *Arctagrostis* Griseb. (weakly supported, PP = 0.64). The subclade of Alopecurinae s. str. (without *Beckmannia*) is supported only according to Bayesian analysis (PP = 0.54, BS unsupported). *Rhizocephalus orientalis* Boiss. is a sister to all other taxa in the Alopecurinae subclade, though only with weak support in Bayesian inference (PP = 0.54, BS unsupported). Part of the sect. *Colobachne* (*A. brevifolius* (G.Westb.) Grossh., *A. glacialis*, *A. textilis*) falls into one clade (PP = 1, BS = 99) and occupies a sister position to the clade containing other species of *Alopecurus* and *Limnas* + *Arctophila* (Rupr.) Andersson + *Dupontia* R.Br. according to Bayesian analysis (PP = 0.70). The clade *Limnas + Arctophila + Dupontia* is very weakly supported (PP = 0.51, BS = 57). It was discovered as the clade that is the sister to the clade-comprising sect. *Alopecurus*, *A. vaginatus* (Willd.) Trin. (sect. *Colobachne*), the sample of *A. myosuroides* (sect. *Pseudophalaris*) from Dagestan Republic, Northern Caucasus, Russia, and sect. *Alopecurium* (PP = 0.86). In addition, *A. brachystachyus* (high- polyploid species from the section *Alopecurus*) is a sister to the clade comprising section *Alopecurium* and does not group with its possible relative, *A. alpinus*. One sample of *A. myosuroides* from Latvia is found in the clade corresponding to the section *Alopecurium*, according to ITS analysis.

We analyzed the marker sequences of 18S rDNA–ITS1–5.8S rDNA obtained via NGS to investigate the hidden multiple hybridization. When the sequence was present in more than 10,000 reads per rDNA pool, we calculated the percentage for them and took these sequences as major. In other cases, when the total quantity of reads was below 10,000, we calculated their percentage for the sequences that occurred in 100 and more reads.

**Figure 4:**
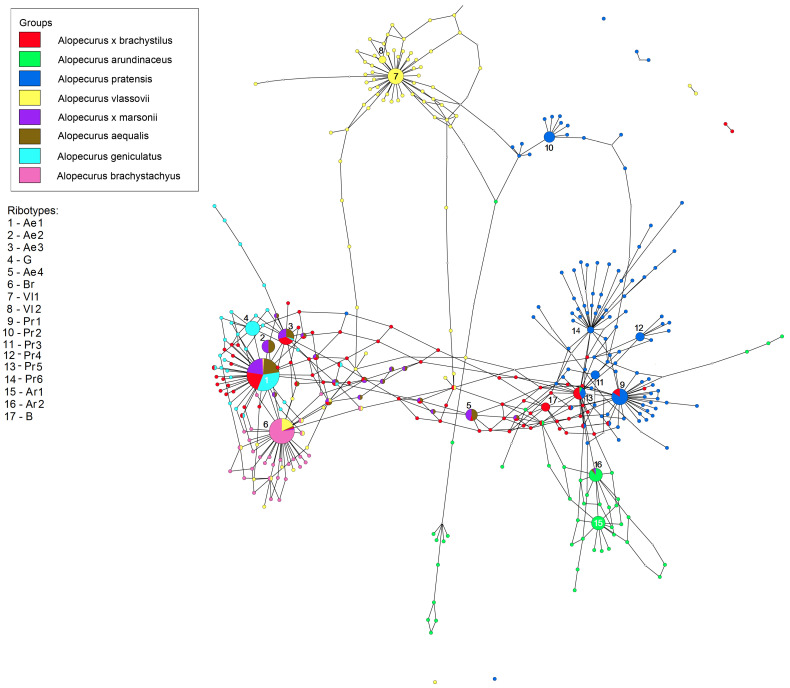
Ribotype network of hybrid species *Alopecurus* × *brachystylus*, *A.* × *marssonii*, their putative parental species and high polyploids *A. vlassovii* and *A. brachystachyus*. The radius of the circles on the ribotype network is proportional to the percent number of reads for each ribotype. Major ribotypes are larger than others and marked with numbers.

**Figure 5:**
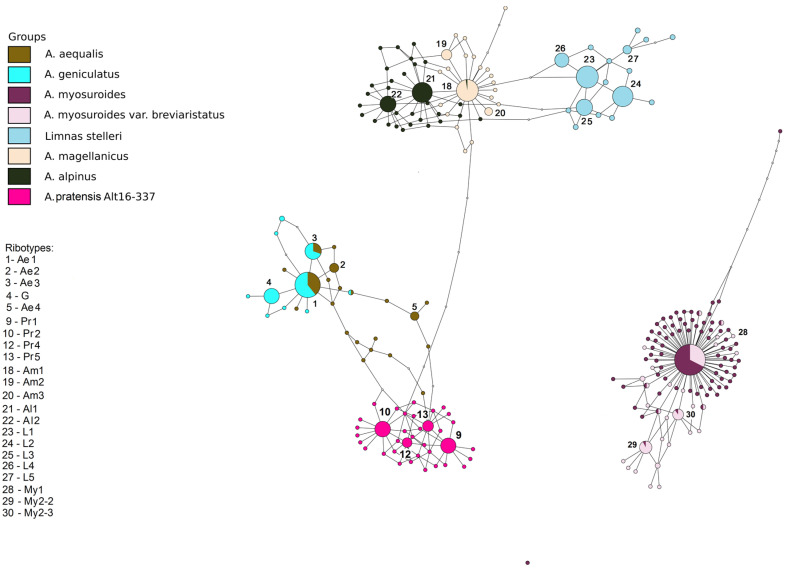
Ribotype network of the sections *Alopecurium*, *Pseudophalaris*, *Alopecurus*, and the genus *Limnas* (*Limnas stelleri*). The radius of the circles on the ribotype network is proportional to the percent number of reads for each ribotype. Major ribotypes are larger than others and marked with numbers.

**Figure 6:**
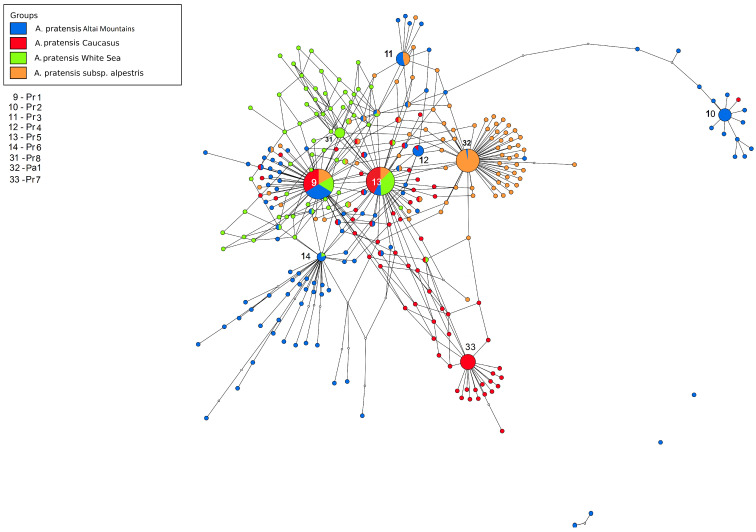
Ribotype network depicting the relationships between different samples of *Alopecurus pratensis.* The radius of the circles on the ribotype network is proportional to the percent number of reads for each ribotype. Major ribotypes are larger than others and marked with numbers.

**Figure 7:**
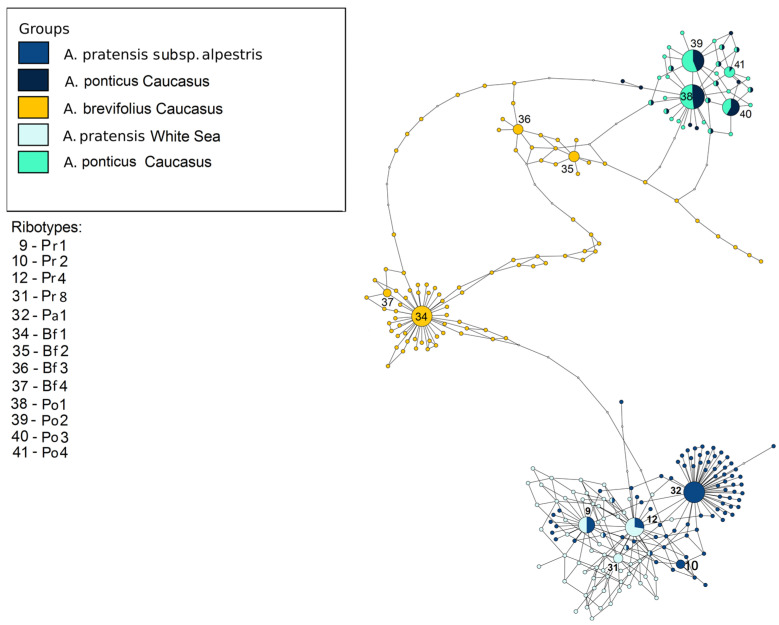
Ribotype network of the section *Alopecurus* (*A. pratensis*) and section *Colobachne*. The radius of the circles on the ribotype network is proportional to the percent number of reads for each ribotype. Major ribotypes are larger than others and marked with numbers.

**Figure 8:**
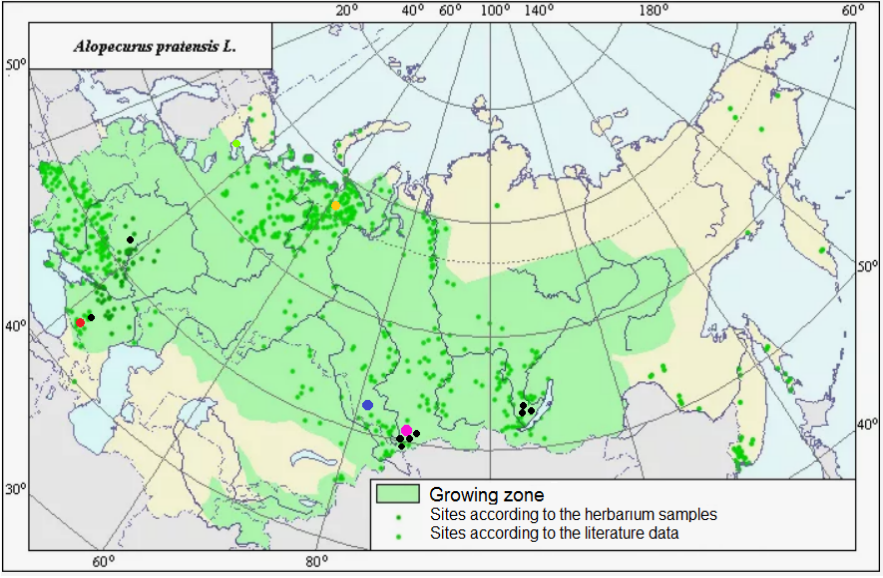
Area of distribution of *Alopecurus pratensis* in Russia and adjacent territories and collection points for our herbarium material. Colored rounds depict collection sites of the samples for NGS analysis; black dots—sites of the samples for chloroplast sequence data. Total species area was taken from http://agroatlas.ru, accessed on 5 March 2024.

The ribotype networks built in TCS 1.21 and visualized in TCSBU are presented in Figure 4, Figure 5, Figure 6, Figure 7 and Figure 8. The first of the networks depicts the origin of hybrid *A.* × *brachystylus* (*A. geniculatus* × *A. pratensis*) and *A.* × *marssoni* (*A. geniculatus* × *A. arundinaceus*) with regard to the parental taxa, e. g., diploid *A. aequalis* (2n = 14) (Figure 4). In addition, we took into analysis *A. vlassovii* Trin.—a high polyploid (2n = 120) of the sect. *Alopecurus*, a relative of *A. alpinus*. We took the main ribotype of *A. aequalis* as the consensus sequence. Looking at the network, we can distinguish three ribotype subnetworks corresponding to the sections *Alopecurium*, *Alopecurus*, and the high-polyploid group (*A. vlassovii*). *Alopecurus aequalis*, a diploid species, has four major ribotypes (in this case, upper 100 reads per rDNA pool) (Figure 4). Its main ribotype, Ae1 (473 reads, 22%), is identical to the main ribotype of *A. geniculatus* (1809 reads, 34%), the main ribotypes of hybrids *A.* × *brachystylus* and *A.* × *marssonii* Hausskn. (5828 reads, 22%, and 465 reads, 22%, respectively). Ribotype Ae1 also has the minor ribotype fraction of *A. vlassovii* and *A. brachystachyus* (below 1%). The second major ribotype of *A. aequalis*, Ae2 (169 reads, 8%), is common with the second major ribotype of *A.* × *marssonii* (164 reads, 8%). Ribotype Ae3 (161 read, 7%) of *A. aequalis* also corresponds to the second major ribotype of *A.* × *brachystylus* (2331 read, 9%) as well as to the third major ribotype of *A.* × *marssonii* (158 reads, 7%), and minor ribotype of *A. geniculatus* (862 reads, 16%). The fourth major ribotype of diploid *A. aequalis*—Ae4 (140 reads, 6%) is placed between “Alopecurium” and “Alopecurus” subnetworks. This ribotype is shared with the fourth major ribotype of hybrid *A.* × *marssonii* (133 reads, 6%) and minor ribotype of *A.* × *brachystylus* (150 reads). The second major ribotype of tetraploid *A. geniculatus* is species-specific (G, 1072 reads, 20%). Surprisingly, the main ribotype of *A. brachystachyus* (Br, 3485 reads, 47%) also belongs to the “Alopecurium” subnetwork. It is also the second major ribotype of *A. vlassovii* (2279 reads, 11%). The main ribotype of *A. vlassovii* is species-specific (Vl1, 5248 reads, 24%) and forms the separate subnetwork along with its derivative, Vl2 (1006 reads, 5%). The second major ribotype of *A. pratensis* unites with this subnetwork (Pr2, 2763 reads, 11%). The main ribotype of *A. pratensis* (Pr1, 5561 read, 21%) groups with “Alopecurus” subnetwork and is homologous to the fifth major ribotype of hybrid *A.* × *brachystylus* (1087 reads, 4%). The third major ribotype of *A. pratensis*, Pr3 (1928 reads, 7%), is specific as well as the fourth (Pr4, 1863 reads, 7%) and the sixth (Pr6, 1049 reads, 4%) ones. Unlike this, the fifth major ribotype of *A. pratensis* (Pr5, 1416 reads, 5%) is identical to the third major ribotype of *A.* × *brachystylus* (2095 reads, 8%) and minor ribotype of *A. arundinaceus* (41 read). Two major ribotypes of *A. arundinaceus*, Ar1 and Ar2 (2864 reads, 18%, and 2504 reads, 15%, respectively), form a separate cluster within the “Alopecurus” subnetwork. The main ribotype Ar1 is specific, and the second major ribotype is shared with the minor ribotypes of *A. aequalis* (75 reads) and *A.* × *marssonii* (73 reads). The fourth major ribotype of *A.* × *brachystylus* (B, 1722 reads, 7%) is species-specific.

The second ribotype network concerns the relationships between section *Alopecurium*, a high-polyploid group of the type section (*A. alpinus*, *A. magellanicus*), section *Pseudophalaris* (*A. myosuroides*) and the genus *Limnas* (Figure 5). The studied species of the section *Alopecurium* do not have the ribotypes of *A. alpinus* and *A. magellanicus* in the rDNA pool. The major ribotypes of *A. alpinus* and *A. magellanicus* (Al and Am, respectively) are mostly species-specific except for the main ribotype of *A. magellanicus* (Am1, 2051 read, 40%) that is shared with the minor ribotype of *A. alpinus* (274 reads). The sample of *A. pratensis* Alt 16-337 has four major ribotypes forming own subnetwork (Pr1, Pr2, Pr5, Pr4) that are identical to the four ribotypes of the *A. pratensis* sample from the previous network. *Limnas stelleri*, a species from the subtribe Alopecurinae, has ribotypes that are close to the *Alopecurus magellanicus*/*A. alpinus* subnetwork. Major ribotypes of this species can be called L. The ribotypes of the samples of *A. myosuroides* have no connection with other samples of *Alopecurus* in this network, according to NGS data. The main ribotype of *A. myosuroides* (My1, 10,044 reads, 56%) is identical with that of *A. myosuroides* var. *breviaristatus* Asch. & Graebn. (3292 reads, 27%). Other major ribotypes (more than 1000 reads in this case) are present only in *A*. *myosuroides* var. *breviaristatus* (My2-2, 1971 read, 12%, My2-3, 1478 reads, 9%). They are common with minor ribotypes of *A. myosuroides.*

The third ribotype network shows the phylogenetic structure of *A*. aggr. *pratensis* (Figure 6). The main ribotype of *A. pratensis* s. str. from Altai Krai, Russia (Pr1, 5561 reads, 21%) is shared with the second major ribotype of *A. pratensis* subsp. *alpestris* (Wahlenb.) Selander (2889 reads, 11%), the second major ribotype of *A. pratensis* from Teberda (4775 reads, 22%), and the second major ribotype of *A. pratensis* from the White Sea (1732 reads, 11%). The second major ribotype of *A. pratensis* from Altai Krai (Pr2, 2763 reads, 11%) is specific. The third major ribotype of *A. pratensis* s. str. (Pr3, 1928 reads, 7%) is common with the minor ribotype of Caucasian *A. pratensis* from Teberda (18 reads). The fourth major ribotype of *A. pratensis*, Pr4 (1863 reads, 7%) is common with fourth major ribotype of *A. pratensis* subsp. *alpestris* (1611, 5%). The fifth major ribotype (Pr5, 1416 reads, 5%) is identical to the main ribotype of *A. pratensis* from Teberda (5359 reads, 24%), the main ribotype of *A. pratensis* from the White Sea (3153 reads, 21%), and the third major ribotype of *A. pratensis* subsp. *alpestris* (1903 reads, 7%) while the sixth major ribotype, Pr6 (1049 reads, 4%) is shared with minor ribotype of *A. pratensis* from the White Sea (49 reads). The third major ribotype of *A. pratensis* from Teberda (Pr7, 3539 reads, 16%) is specific as well as the third major ribotype of *A. pratensis* from the White Sea (Pr8, 1041 read, 7%), and the main ribotype of *A. pratensis* subsp. *alpestris* (Pa1, 9301, 37%) is common with minor fraction of *A. pratensis* (547 reads, below 1%).

The next picture describes the relationships between the studied samples of the sections *Alopecurus* (*A. pratensis* s. l.) and *Colobachne* (Figure 7). ITS1 sequences of *A. pratensis* s. str. samples from the White Sea and *A. pratensis* subsp. *alpestris* obtained by NGS group separately from those of *A. ponticus*. The main ribotype of *A. pratensis* from the White Sea is the same as the main ribotype of *A. pratensis* subsp. *alpestris* (Pr1). The main ribotype of *A. pratensis* subsp. *alpestris* (Pa1, 9301, 37%) is related to the ribotypes of *A. pratensis* but is specific on this scheme. The fourth major ribotype of *A. pratensis* subsp. *alpestris* (1611, 5%) belongs to the group Pr and is identical to the ribotype Pr4 (see above). The ribotype structure of the species of the sect. *Colobachne* is diverse. *A. brevifolius* sample from Teberda (T89) forms the peculiar subnetwork that is only distantly related to the sect. *Alopecurus*. Its ribotypes, Bf1–Bf4 are specific. Ribotypes of two samples of *A. ponticus* from Teberda (T57, T59) are almost identical except for the fourth major ribotype of *A. ponticus* T59 (Po4) that is shared with the minor ribotype of *A. ponticus* T57.

## 3. Discussion

When a species has substantially discordant positions between the cpDNA and nrDNA phylogenies, there is a possibility that the species may be a hybrid [11,12,13,14]. Moreover, intraspecific genome polymorphism is widely used in research for interspecific and, in some cases, intraspecific phylogenetic reconstructions, especially in cases when introgression is involved [15,16,17,18]. For example, some genera of the tribe Triticeae Dumort. (Poaceae) appeared to be of intergeneric origin themselves [19,20,21]. Molecular phylogenetic analysis of different gene sets that allowed to trace possible hybridization events proposed generic names based on the genome combinations that the species has [22]. Other works presented new data of multiple polyploid origin of many *Hordeum* L. species (sometimes auto- and allopolyploid species were in the same aggregate) [21].

The genus *Alopecurus* belongs to the subtribe Alopecurinae (tribe Poeae s. l.), which is known for widespread hybridization. Molecular phylogenetic studies of the genus showed polyphyletic placement of the previously described sections *Alopecurus* and *Colobachne* [7,23]. Some replacements were also made on the species level that can have, in fact, different explanations [7,23]. Researching the ribotype composition of *Alopecurus* and some taxa from allied genera, we found many ribotypes due to the active hybridization and high polyploidy in the subtribe Alopecurinae.

In the genus *Alopecurus*, four nothospecies were described that are usually sterile and rather rare [2]. We analyzed two of them, *Alopecurus* × *brachystylus* and *A.* × *marssonii*. *A.* × *brachystylus* is an intersectional hybrid according to taxonomists [24,25]. One of the putative parent species, the Euro–Mediterranean–South Asian *A. geniculatus*, belongs to the section *Alopecurium*, the members of which are distinguished by their characteristic narrow cylindrical panicles and geniculate stems. The second parent taxon, the Euro–Siberian–Central Asian *A. pratensis*, belongs to the type section, which includes species with predominantly wide cylindrical or ellipsoidal panicles and straight stems. Parental species, growing in the same habitats, can hybridize, usually forming sterile offspring [26,27]. *A.* × *brachystylus* was not previously studied by molecular phylogenetic methods. Our data confirm this statement but add some details. On the maternal side, *A.* × *brachystylus* probably originated from *A. geniculatus* (Figure 5). Our NGS analysis shows the presence of *A. pratensis* ribotypes and ribotypes that are common with diploid *A. aequalis* (also belonging to the sect. *Alopecurium*). Tetraploid *A. geniculatus* (2n = 28), in its turn, could originate from diploid *A. aequalis* and an unknown diploid progenitor (Figure 4). The second possible interpretation is that *A. geniculatus* has the second ribotype that passed the stages of post-hybridization transformation. Thus, the hybrid genome of *A.* × *brachystylus* has only *A. aequalis* ribotypes from an *A. geniculatus* parent. *A*. × *marssonii* is a hybrid between *A. geniculatus* and Predominantly Euro–Siberian–Caucasian–Central Asian meadow–coastal weakly halophilic species *A. arundinaceus.* Ribotype structure of *A*. × *marssonii* indicates its origin from *A. geniculatus* (hybrid with *A. aequalis* as parental species). *A. arundinaceus*-related ribotypes occurred only in a minor fraction of *A*. × *marssonii.* But, surprisingly, a minor ribotype fraction that is common with A. *arundinaceus* was also found in *A. aequalis* ribotypes (Figure 4). This unexpected fact can point at the cases of introgression that took place in in the fairly distant past. All trees built on the sequence data, obtained by the Sanger method, clearly show distinction of the sect. *Alopecurium*.

South Siberian mountain species *A. vlassovii*, which belongs to the high polyploid group of the sect. *Alopecurus* (*A*. aggr. *borealis*), has poly- and aneuploid chromosome numbers, 2n = ca. 98–130, ca. 120, and ca. 150, without predominance of any one of them [28,29,30]. According to the NGS data, *A. vlassovii* has two subgenomes that correspond to the *A. borealis* group and *A. brachystachyus* (being close to the sect. *Alopecurium*) (Figure 4). High polyploidy of *A. vlassovii* is clearly a result of multiple hybridization and introgression within the group and probably between the different sections. *A. vlassovii* can be a member of a peculiar introgressive–hybridization complex of species [31] belonging to the affinity group of *A. alpinus*. *A. vlassovii* is possible hybrid of *A. alpinus* s. l. and *A. brachystachyus*. The latter species, according to *rbc*L and *mat*K sequence analysis, has different maternal taxa. The majority of *A. brachystachyus* are relatives of *A. alpinus* s. l. and other high-polyploid mountain species but some samples occupy an uncertain position (Figure 1). ITS data obtained by the Sanger method, along with the NGS data, show the relationship between *A. brachystachyus* and *A. aequalis* + *A. geniculatus*.

R. Soreng et al. [32] synonymized the Antarctic species *Alopecurus magellanicus*, Holarctic mountain-tundra species *A. alpinus*, and *A. borealis*, North Pacific mountain-meadow *A. stejnegeri* Vasey and endemic to the Ural Mountains, weakly halophilic *A. glaucus.* In our opinion, we cannot accept such a broad interpretation of *A. magellanicus*, since these undoubtedly closely related species nevertheless differ in both morphological and molecular genetic characteristics. A comparison of herbarium material of these species with specimens of *A. magellanicus* (South Georgia and the South Sandwich Islands) did not reveal absolute similarity between them. In contrast, *A. magellanicus* has its own species-specific characters not found in other closely related species. For example, *A. magellanicus* has larger spikelets (4–5 mm long) than *A. alpinus*, less abundant pubescence of the glumes, more developed awns, and the presence of a membranous border at the tips of the lemmas. Subantarctic *A. magellanicus* and Arctic *A. alpinus* have their own specific ribotypes (Figure 5). Only a minor ribotype fraction of *A. alpinus* is shared with *A. magellanicus*. *A. magellanicus* is a close relative of *A. alpinus* and *A. brachystachyus* according to ndhF sequence analysis. *A. alpinus*, as an ancestor of *A. magellanicus*, could spread across Cordilleras, this being the classic example of interpolar disjunction (see also [33]).

*Alopecurus myosuroides*, belonging to the sect. *Pseudophalaris*, stands apart from other species of the genus. The main distinction of *A. myosuroides* is its annual life form and the winged keel on its glumes. We see that *A. myosuroides* also stands separately according to the molecular phylogenetic data (Figure 1, Figure 2, Figure 3, Figure 4 and Figure 6). Morphological separateness along with distinction of the sequences can point at possible generic specificity of *A. myosuroides*. But one sample of *A. myosuroides* from Latvia falls into a clade with *A. aequalis* and *A. geniculatus* (sect. *Alopecurium*). This unusual position can reflect the ancient homoploid hybridization.

Another interesting fact is the position of *Limnas*. The genus *Limnas* is a member of subtribe Alopecurinae and is distinguished by more or less loose panicles and leathery-membranous glumes [2]. There are two endemic species of *Limnas* in Russia: Eastern Siberian *Limnas stelleri* and Eastern Siberian/Far Eastern *Limnas malyschevii*. It is interesting that *Limnas stelleri* turned out to be more or less closely related with North American *Alopecurus alpinus* and Subantarctic *A. magellanicus* (Figure 5). The close relationship of *Limnas* and *Alopecurus* species was previously identified by Soreng [23], using ITS data obtained by the Sanger method (Figure 3). The second species, *L. malyschevii*, is weakly related to the clade *Dupontia* + *Arctophila* by ITS data and is sister to *Alopecurus* clade by *ndh*F sequences (Figure 2). Such unusual placement of two *Limnas* species could be due to the intergeneric reticulation or even polyphyletic origin of the genus.

Molecular phylogenetic data often help in distinguishing different species in one large geographically heterogeneous complex. For example, analysis of chloroplast and nuclear (ITS) genes revealed a different position of Malaysian samples of *Spiranthes sinensis* (Pers.) Ames (Orchidaceae Juss.) compared with other East Asian samples [34]. They were considered as crypto-hybrids [34]. In grasses (Poaceae), even samples of *Hyalopoa pontica* (Balansa) Tzvelev from different but closely located gorges of Greater Caucasus (Teberda, Carachay-Cherkessia Republic) can fall into different clades [35]. *Alopecurus pratensis* has almost a worldwide area, occurring in many non-tropical areas. It is tetraploid with 2n = 28 [2]. It forms a group of related taxa: *A. pratensis* s. str., *A. pratensis* subsp. *alpestris*, *A. arundinaceus*, and *A. brachystachyus*. *A. pratensis* subsp. *alpestris* is an Arctic or hypoarctic plant with glaucous tinge of ligules and glaucous leaves. Our NGS data confirm that *A. pratensis* subsp. *alpestris* can be can be distinguished as a separate species because it has specific main ribotype (Figure 6). In addition, *A. pratensis* from Northern Caucasus (Teberda) and *A. pratensis* from the White Sea coast have peculiar major ribotypes but not main one. It can be related with the geographical variability of *A. pratensis* s. l. (Figure 8). In addition, geographical variability was detected by chloroplast sequence data (*rbc*L and *mat*K). Samples from Stavropol Krai and Altai Republic differ from those from Irkutsk Oblast and other samples from Altai Republic (Figure 1). It is possible that *Alopecurus pratensis* is heterogeneous by the maternal line. *A. brachystachyus*, on the contrary, has different affinity according to the chloroplast sequence data; it is closer to the Arctic and Sub-Arctic high polyploid species but also can have an *A. pratensis*-like maternal genome (Figure 1, see above).

**Figure 9:**
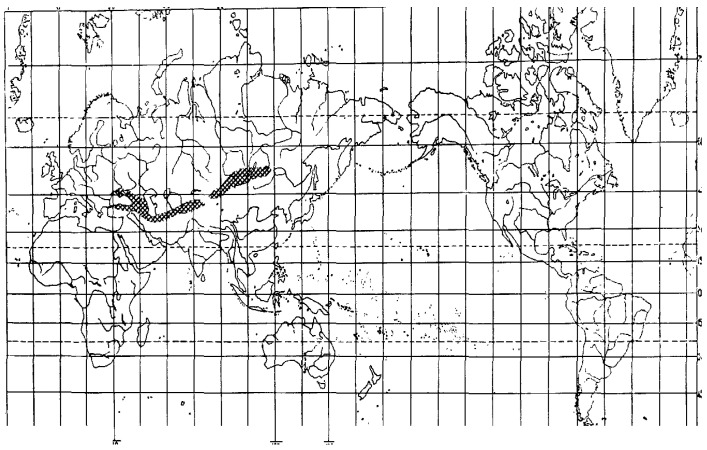
Area of distribution of the *Vaginatae* group (=section *Colobachne*) [36].

Species from the section *Colobachne* differ from the other *Alopecurus* members by wide ellipsoid or shortly cylindrical panicles and by predominant presence of palea [2,24]. The center of their diversity is Ancient Mediterranean and Caucasus region ([36], Figure 9).

They form polyploid range from 2n = 14 [30] to 2n = 56 [37]. We need to note that *A. brachystachyus* (sect. *Alopecurus*) was previously treated as the species of the *Vaginatae* group (=sect. *Colobachne*) as well [36]. Molecular phylogenetic analysis clearly confirms distinction of this section from high-polyploid relatives of *A. alpinus* and from *A. brachystachyus* and comparative unity of the section *Colobachne* on the maternal side (*rbc*L and *mat*K, Figure 1 and Figure 2). At the same time, the nrDNA of the *Colobachne* species indicates probable hybridizations: *A. vaginatus* falls into a clade with *A. alpinus* and allied taxa (Figure 3) and is distant from other members of the sect. *Colobachne*. Additionally, the section *Colobachne* (excl. *A. vaginatus*) is rather distant form all other *Alopecurus* members based on ITS sequences (Figure 3). According to NGS data, studied species of the section *Colobachne* are fairly distinct from each other and do not form common ribotypes (Figure 7). We can assume that species of the sect. *Colobachne* probably arose from intersectional hybridization but are rather stabile themselves.

The position of two genera, *Beckmannia* and *Rhizocephalus*, remains controversial. Earlier, these genera were placed near the genus *Alopecurus* in the tribe Phleeae [24,38]. These genera were placed in the special subtribe Beckmanniinae. Our data present *Beckmannia* as the genus allied to the subtribe Alopecurinae that is closer to the latter on the chloroplast trees (Figure 1 and Figure 2). *Rhizocephalus orientalis* occupies an uncertain position being sister to Alopecurinae only by Bayesian method according to the ITS data (PP = 0.54) (Figure 3).

Thus, our data prove developed hybridization in the subtribe Alopecurinae, not only in the sterile nothospecies. The degree of introgressive hybridization processes varies depending on the sections and groups; e.g., some *Alopecurus* species do not hybridize with closely related taxa according to NGS data but have plastid DNA from different sections. We tend to re-establish the high polyploid group of *Alopecurus* previously named as the group *Alpinae* [36] that now is placed within the sect. *Alopecurus*. The genus *Limnas* is probably polyphyletic.

## 4. Materials and Methods

For our molecular phylogenetic analysis, we took species from all sections of the genus *Alopecurus*, paying special attention to the potential hybrid taxa. We included also species from other genera of the subtribe Alopecurinae s. l.: *Beckmannia*, *Limnas*, *Rhizocephalus* as well. We analyzed chloroplast sequences of the region *mat*K of 53 samples of 13 species, *rbc*L in 61 samples of 12 species, *ndh*F of 19 samples of 11 species, and 27 ITS sequences of 16 species (all obtained by Sanger method). Then, we concatenated *rbc*L and *mat*K sequences of the studied species when there were both sequences of the same species. The sequences of *rbc*L + *mat*K regions were presented for 48 samples of 10 species. We paid more attention to the hybrid species and polyploid species that can have some differences along their habitat. Information about the species studied via Sanger method is given in Table 1. For NGS analysis, we used 23 species of the subtribe Alopecurinae. The list of studied species is presented in Table 2.

In the case of 10,000 reads per rDNA pool, we assumed the ribotypes more than 1000 reads per rDNA pool to be major. When the total quantity of reads was below 10,000, we took the sequences of ribotypes that occurred in 100 or more reads as menomic DNA, which was extracted from leaf material with the aid of a Qiagen Plant Mini Kit (Qiagen Inc., Hilden, Germany), according to the instruction manual. The fragments were amplified and sequenced at the Center for Shared Use “Genomic Technologies, Proteomics, and Cell Biology” of the All-Russian Research Institute of Agricultural Microbiology on an Illumina Platform MiSeq. PCR was performed in 15 μL of the reaction mixture containing 0.5–1 unit of activity of Q5^®^ High-Fidelity DNA Polymerase (NEB, Ipswich, MA, USA), 5 pM of forward and reverse primers, 10 ng of DNA template, and 2 nM of each dNTP (Life Technologies, ThermoScientific, Waltham, MA, USA). The fragments were amplified under the following conditions: initial denaturation at 94 °C for 1 min, followed by 25 cycles of 94 °C for 30 s, 55 °C for 30 s, 72 °C for 30 s, and a final elongation 72 °C for 5 min using ITS 1P [39] and ITS 2 [40] primers. PCR products were then purified according to the Illumina recommended method using AMPureXP (Beckman Coulter, Indianapolis, IN, USA). The libraries for sequencing were prepared according to the manufacturer’s MiSeq Reagent Kit Preparation Guide (Illumina) (http://web.uri.edu/gsc/files/16s-metagenomic-library-prep-guide-15044223-b.pdf (accessed on 6 September 2022)). They were sequenced on an Illumina MiSeq instrument (Illumina, San Diego, CA, USA) using a MiSeq^®^ ReagentKit v3 (600 cycles) with double-sided reading (2 × 300 n) following the manufacturer’s instructions. The sequences were trimmed with Trimmomatic [41], included in Unipro Ugene [42] as follows: PE reads, sliding window trimming with size 4, quality threshold 12, and minimal read length 130. Further, paired marker sequences were combined, dereplicated, and sorted into the ribotypes by vsearch 2.7.1 [43]. The resulting sequences represent ribotypes with certain frequency in the whole genome pool. These sequences were analyzed by TCS 1.21 [44]. The network built by algorithms of statistic parsimony was visualized and processed in TCSBU [45].

Forward and reverse sequences of ITS and chloroplast regions obtained by Sanger method were observed in Chromas Lite 2.1 (https://technelysium.com.au/wp/chromas/, accessed on 1 October 2021) and concatenated in MEGA XI [46]. The sequencing was performed according to the standard protocol provided with a BigDyeTM Terminator Kit ver. 3.1 set of reagents on the sequencer ABI PRIZM 3100 sequencer at the Center for the collective use of scientific equipment “Cellular and molecular technologies for the study of plants and fungi” of the Komarov Botanical Institute, St. Petersburg. They were aligned by Muscle [47] implemented in MEGA XI [46]. Evolutionary models were computed using jModelTest 2.1.10 [48]. For the ITS dataset, we obtained GTR + I + G, for *mat*K TPM3uf + G, for *ndh*F, the evolutionary model was TVM + I + G, and for *rbc*L we computed TVM + G. Bayesian inference was performed using Mr. Bayes 3.2.2 [49] as follows: 3–5 million of a generation, sampling trees every 100 generations, and the first 25% trees were discarded as burn-in. ML analysis was conducted by iqtree 1.6.12 (http://www.iqtree.org/, accessed 5 March 2023) under the fast bootstrap option, 1000 generations. The resulting trees combined Bayesian and ML data. The first index was posterior probability, and the second was a bootstrap index.

## Figures and Tables

**Table 1:** Sequences of the species of subtribe Alopecurinae and related subtribes obtained in the present study by the Sanger method and their numbers in GenBank.

Species	Country of Origin	Genbank Number, rbcL	Genbank Number, matK	Genbank Number, ndhF	Genbank Number, ITS
*Alopecurus aequalis*	Russia: Yakutia Republic				PP048962
*Alopecurus aequalis*	Russia: Kurgan Oblast				PP048965
*Alopecurus aequalis*	Russia: Irkutsk Oblast				PP048966
*Alopecurus aequalis*	Russia: Altai Republic	PP060934	OR997845		
*Alopecurus aequalis*	Russia: Altai Republic	PP060935	OR997846		
*Alopecurus aequalis*	Russia: Irkutsk Oblast	PP060936	OR997847		
*Alopecurus aequalis*	Russia: Irkutsk Oblast	PP060937			
*Alopecurus aequalis*	Russia: Altai Republic	PP060938	OR997848		
*Alopecurus aequalis*	Russia: Irkutsk Oblast		OR997849		
*Alopecurus aequalis*	Russia: Altai Republic	PP060939	OR997850		
*Alopecurus aequalis*	Russia: Irkutsk Oblast	PP060940			
*Alopecurus aequalis*	Russia: Irkutsk Oblast	PP060941	OR997851		
*Alopecurus aequalis*	Russia: Leningrad Oblast	PP060942	OR997852		PP048963
*Alopecurus aequalis*	Russia: Irkutsk Oblast	PP060943			
*Alopecurus aequalis*	Russia: Krasnodar Krai	PP060944	OR997853		PP048964
*Alopecurus aequalis*	Russia: Irkutsk Oblast		OR997854		
*Alopecurus aequalis*	Russia: Altai Republic	PP060945	OR997855		
*Alopecurus alpinus*	Russia: Altai Republic			PP053715	PP048961
*Alopecurus alpinus*	Russia: Tuva Republic			PP053716	
*Alopecurus alpinus*	Russia: Altai Republic	PP060946	OR997856		
*Alopecurus alpinus*	Russia: Altai Republic	PP060947	OR997857		
*Alopecurus alpinus*	Russia: Altai Republic	PP060948	OR997858		
*Alopecurus alpinus*	Russia: Altai Republic	PP060949	OR997859		
*Alopecurus alpinus*	Russia		OR997860		
*Alopecurus alpinus*	Russia: Altai Republic	PP060950	OR997861		
*Alopecurus alpinus*	Russia: Altai Republic	PP060952	OR997862		
*Alopecurus alpinus*	Russia: Altai Republic	PP060953	OR997863		
*Alopecurus alpinus*	Russia: Altai Republic	PP060954	OR997864		
*Alopecurus alpinus*	Russia: Altai Republic	PP060955	OR997865		
*Alopecurus arundinaceus*	Russia: Yakutia Republic			PP053720	
*Alopecurus arundinaceus*	Russia: Irkutsk Oblast	PP060956	OR997866		
*Alopecurus arundinaceus*	Russia	PP060957	OR997867		
*Alopecurus arundinaceus*	Russia: Buryatia Republic	PP060958	OR997868		
*Alopecurus arundinaceus*	Russia	PP060959	OR997869		
*Alopecurus arundinaceus*	Russia	PP060960	OR997870		
*Alopecurus arundinaceus*	Russia: Irkutsk Oblast	PP060961	OR997871		
*Alopecurus arundinaceus*	Russia: Irkutsk Oblast	PP060962	OR997872		
*Alopecurus arundinaceus*	Russia: Irkutsk Oblast	PP060963	OR997873		
*Alopecurus brachystachyus*	Russia: Irkutsk Oblast	PP060964	OR997874	PP053718	
*Alopecurus brachystachyus*	Russia: Irkutsk Oblast	PP060965			
*Alopecurus brachystachyus*	Russia: Irkutsk Oblast	PP060966	OR997875		
*Alopecurus brachystachyus*	Russia	PP060967			
*Alopecurus brachystachyus*	Russia	PP060968	OR997876		
*Alopecurus brachystylus*	Russia: Irkutsk Oblast		OR997877		
*Alopecurus brachystylus*	Russia: Irkutsk Oblast		OR997878		
*Alopecurus glacialis*	Russia: Kabardino-Balkaria	PP060969			
*Alopecurus glacialis*	Russia: Kabardino-Balkaria	PP060970	OR997879		PP048960
*Alopecurus laguroides*	Russia: Dagestan Republic	PP060971	OR997880		
*Alopecurus myosuroides*	Russia: Stavropol Krai	PP060972			
*Alopecurus myosuroides*	Russia: Stavropol Krai	PP060973	OR997881		
*Alopecurus myosuroides*	Russia: Stavropol Krai	PP060974	OR997882		
*Alopecurus myosuroides*	Russia: Stavropol Krai	PP060975			
*Alopecurus myosuroides*	Russia: Stavropol Krai	PP060976			
*Alopecurus pratensis*	Russia: Altai Republic			PP053717	
*Alopecurus pratensis*	Russia: Altai Republic	PP060977	OR997883		
*Alopecurus pratensis*	Russia: Altai Republic	PP060978	OR997884		
*Alopecurus pratensis*	Russia	PP060979	OR997885		
*Alopecurus pratensis*	Russia: Irkutsk Oblast	PP060980	OR997886		
*Alopecurus pratensis*	Russia	PP060981	OR997887		
*Alopecurus pratensis*	Russia: Irkutsk Oblast	PP060982	OR997888		
*Alopecurus pratensis*	Russia: Irkutsk Oblast	PP060983	OR997889		
*Alopecurus pratensis*	Russia	PP060984			
*Alopecurus pratensis*	Russia: Irkutsk Oblast	PP060985	OR997890		
*Alopecurus pratensis*	Russia: Altai Republic	PP060986	OR997891	PP053719	
*Alopecurus pratensis*	Russia: Altai Republic	PP060987	OR997892		
*Alopecurus pratensis*	Russia: Lipetsk Oblast	PP060988	OR997893		
*Alopecurus pratensis*	Russia: Stavropol Krai	PP060989			
*Alopecurus pratensis*	Russia	PP060990	OR997894		
*Alopecurus ponticus*	Russia: Stavropol Krai	PP060991	OR997895		
*Alopecurus ponticus*	Russia: Karachay-Cherkessia	PP060951			
*Alopecurus ponticus*	Russia: Karachay-Cherkessia	PP060992	OR997896		
*Alopecurus textilis*	Armenia				PP048958
*Alopecurus textilis*	Armenia				PP048959
*Beckmannia syzigachne*	Russia: Altai Republic			PP053722	
*Beckmannia syzigachne*	Russia: Altai Republic	PP060993	OR997897		
*Limnas malyschevii*	Russia: Yakutia Republic			PP053721	
*Limnas stelleri*	Russia: Khakassia Republic	PP060994			
*Milium effusum*	China			PP053726	
*Milium effusum*	China			PP053727	
*Phleum alpinum*	Tajikistan				PP048954
*Phleum alpinum*	Kazakhstan			PP053724	
*Phleum phleoides*	Russia: Altai Republic				PP048955
*Phleum phleoides*	Russia: Altai Krai				PP048956
*Phleum phleoides*	Russia: Altai Republic				PP048957
*Phleum pratense*	Russia: Ryazan Oblast			PP053723	
*Phleum pratense*	Latvia			PP053725	

**Table 2:** Summary of the Alopecurinae species used in the present NGS study and their major ribotypes.

Species	Sample ID	Country of Origin	Accession Number in Genbank Database	Total Number of Reads	Ribotype Symbol	Number of Reads	% from the Total Number of the Reads
*Alopecurus aequalis*	M36	Russia: Altai Krai, Mamontovsky District	PP056744–PP056764	2173	Ae1	473	22
					Ae2	169	8
					Ae3	161	7
					Ae4	140	6
*Alopecurus alpinus*	L22	Canada: Franklin district, Banks Island	PP056764–PP054539	10,709	Al1	3709	35
					Al2	2396	22
*Alopecurus arundinaceus*	22	Russia: Khakassia Republic	PP056966–PP057011	16,316	Ar1	2864	18
					Ar2	2504	15
*Alopecurus brachystachyus*	M13	Russia: Zabaykalsky Krai	PP057607–PP057638	7373	Br	3485	47
*Alopecurus* × *brachystylus*	21	Russia: Novgorod Oblast	PP056765–PP056849	26,133	Ae1	5828	22
					Ae3	2331	9
					Pr5	2095	8
					B	1722	7
					PR1	1087	4
*Alopecurus brevifolius*	L21	Russia: Karachay-Cherkessia, Teberda	PP057450–PP057540	20,957	Bf1	7380	35
					Bf2	1879	9
					Bf3	1643	8
					Bf4	1124	5
*Alopecurus geniculatus*	L18	Ukraine: Lviv Oblast	PP056850–PP056879	5263	Ae1	1809	34
					G	1072	20
*Alopecurus magellanicus*	L14	South Georgia	PP056879–PP057291	5142	Am1	2051	40
					Am2	447	9
					Am3	235	5
*Alopecurus × marssonii*	M11	Finland	PP057250–PP057269	1880	Ae1	465	22
					Ae2	164	8
					Ae3	158	7
					Ae4	133	6
*Alopecurus myosuroides*	20	Russia: Dagestan Republic	PP054402–PP054507	18,721	My1	10,044	56
*Alopecurus myosuroides* var. *breviaristatus*	L16	Russia: Krasnodar Krai	PP057377–PP057405	12,275	My1	3292	27
					My2-2	1971	12
					My2-3	1478	9
*Alopecurus ponticus*	L20	Russia: Karachay-Cherkessia, Teberda	PP057430–PP057449	9229	Po1	2234	24
					Po2	1691	18
					Po3	1319	14
*Alopecurus ponticus*	M8	Russia: Karachay-Cherkessia, Teberda	PP057577–PP057606	13,553	Po1	3562	26
					Po2	3143	23
					Po3	1406	10
					Po4	1142	8
*Alopecurus pratensis*	23	Russia: Altai Krai	PP057012–PP057116	26,172	Pr1	5561	21
					Pr2	2763	11
					Pr3	1928	7
					Pr4	1863	7
					Pr5	1416	5
					Pr6	1049	4
*Alopecurus pratensis*	M5	Russia: Karachay-Cherkessia, Teberda	PP057117–PP057186	22,148	Pr5	5359	24
					Pr1	4775	21
					Pr7	3539	16
*Alopecurus pratensis*	M6	Russia: Arkhangelsk Oblast, Solovetsky Islands	PP057187–PP057249	15,362	Pr5	3153	21
					Pr1	1732	11
					Pr8	1041	7
*Alopecurus pratensis*	L30	Russia: Altai Republic	PP057541–PP057576	14,946	Pr1	2965	20
					Pr2	2863	19
					Pr5	1485	10
					Pr4	1228	8
*Alopecurus pratensis* subsp. *alpestris*	L15	Russia: Komi Republic	PP057292–PP057376	25,290	Pa1	9301	37
					Pr1	2889	11
					Pr5	1903	7
					Pr4	1611	5
*Alopecurus vlassowii*	25	Russia: Altai Republic	PP056880–PP056965	21,628	Vl1	5248	24
					Br	2279	11
					Vl2	1006	5
*Limnas stelleri*	M17	Russia: Irkutsk Oblast	PP057639–PP057654	1704	L1	364	21
					L2	303	18
					L3	195	11
					L4	145	9
					L5	80	5

## Data Availability

Data are contained within the article and Supplementary Materials.

## Supplementary Materials

The following supporting information can be downloaded at: https://www.mdpi.com/article/10.3390/plants13070919/s1, Table S1. *rbc*L sequences used in our analysis. Table S2. matK sequences used in our analysis. Table S3. *ndh*F sequences used in our analysis. Table S4. ITS sequences used in our analysis. Table S5. Primary structure of the major ribotypes of the genus *Alopecurus* and *Limnas stelleri* obtained by NGS. Numbers show the position in the alignment of the major ribotypes. D is a deletion. Table S6. Aligned ribotype sequences of the hybrid species *Alopecurus* × *brachystylus*, *A.* × *marssonii*, their putative parental species and high polyploids *A. vlassovii* and *A. brachystachyus*. Table S7. Aligned ribotype sequences of species of the sections *Alopecurium*, *Pseudophalaris*, *Alopecurus*, and the genus *Limnas* (*Limnas stelleri*). Table S8. Aligned ribotype sequences of different samples of *Alopecurus pratensis* (including *A. pratensis* subsp. *alpestris*). Table S9. Aligned ribotype sequences of *A. pratensis* sample (sect. *Alopecurus*) and species of the section *Colobachne*. Figure S1. Phylogenetic tree of the subtribe Alopecurinae and related species according to the *rbc*L sequence data. The first index on the branch is the posterior probability in Bayesian inference, the second is the bootstrap index obtained by Maximum Likelihood algorithm. When only one index is shown on the branch it is the posterior probability. Figure S2. Phylogenetic tree of the subtribe Alopecurinae and related species according to the *mat*K sequence data. The first index on the branch is the posterior probability in Bayesian inference, the second is the bootstrap index obtained by Maximum Likelihood algorithm. When only one index is shown on the branch it is the posterior probability.
