# Supplementary figures and images for: Hybridization in the Subtribe Alopecurinae Dumort. (Poaceae) According to Molecular Phylogenetic Analysis: Different Ploidy Level Tells Different Origin of the Groups
Source: Plants (Basel). 2024 Mar 22;13(7):919. doi: 10.3390/plants13070919 (PMC11013341; doi:10.3390/plants13070919)

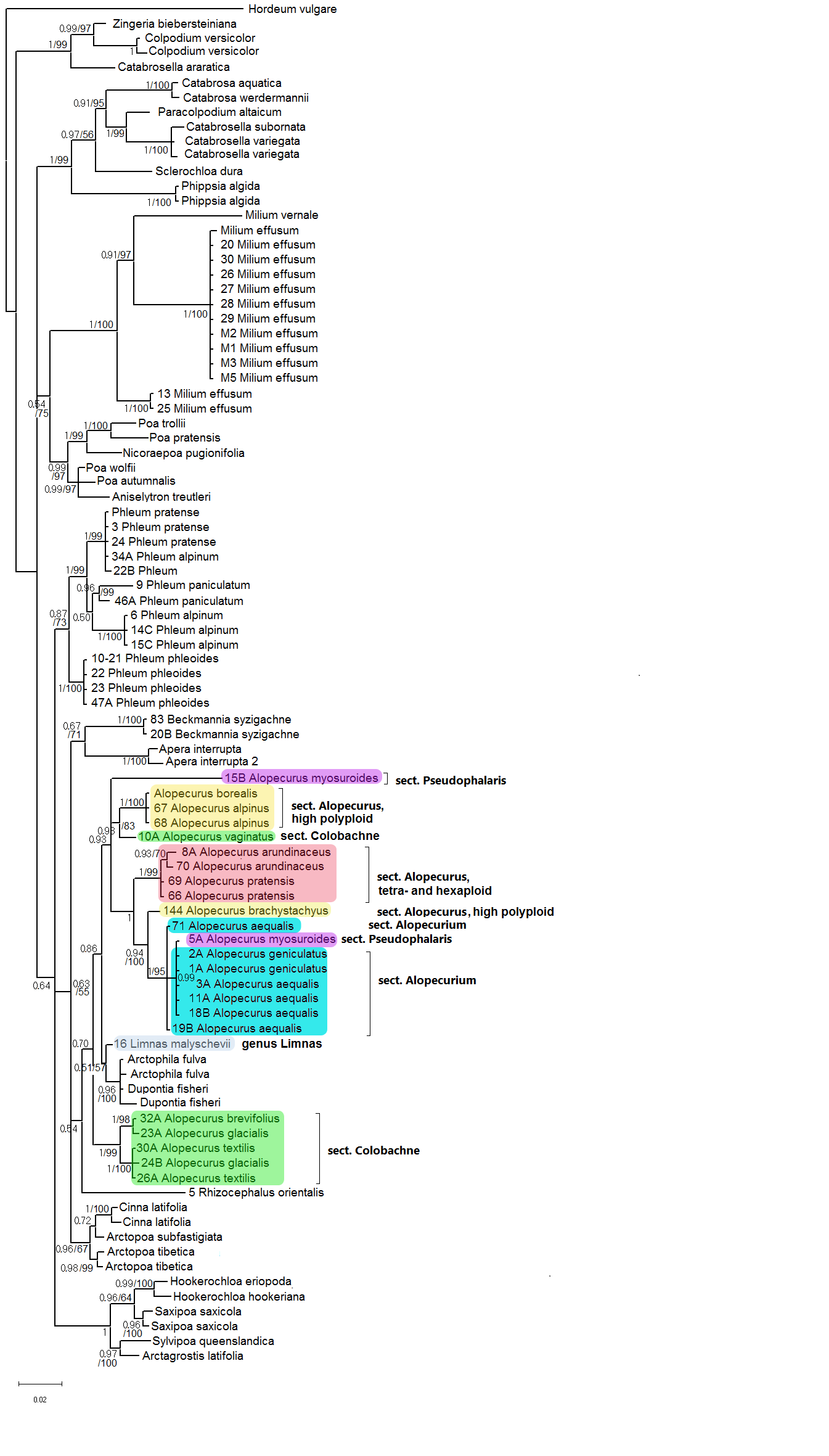

Supplement: Supplementary file 1 [file plants-13-00919-s001.zip › Alopecurus_ITS_colored_figure S3.tif]

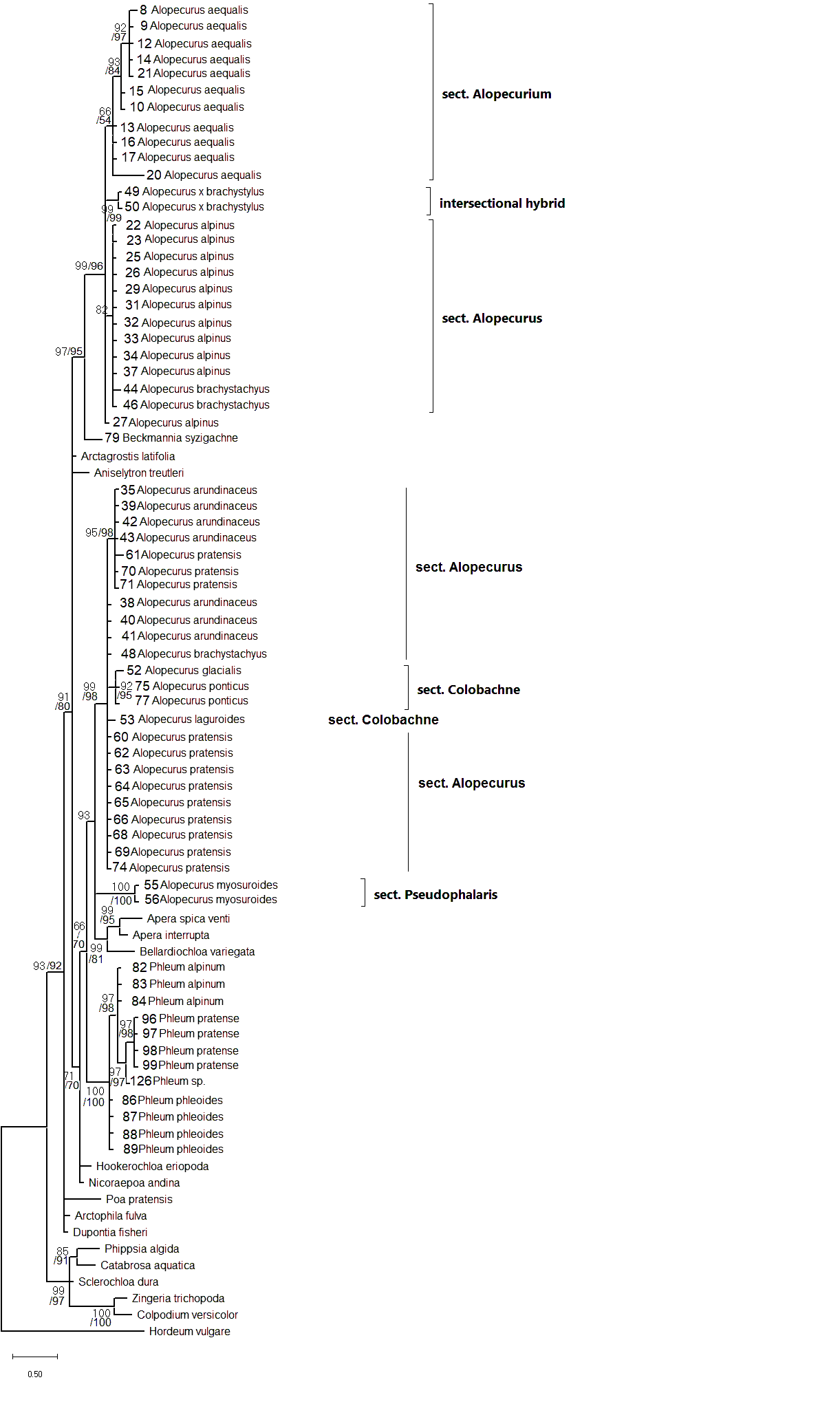

Supplement: Supplementary file 1 [file plants-13-00919-s001.zip › Alopecurus_matK_figure S2.bmp]

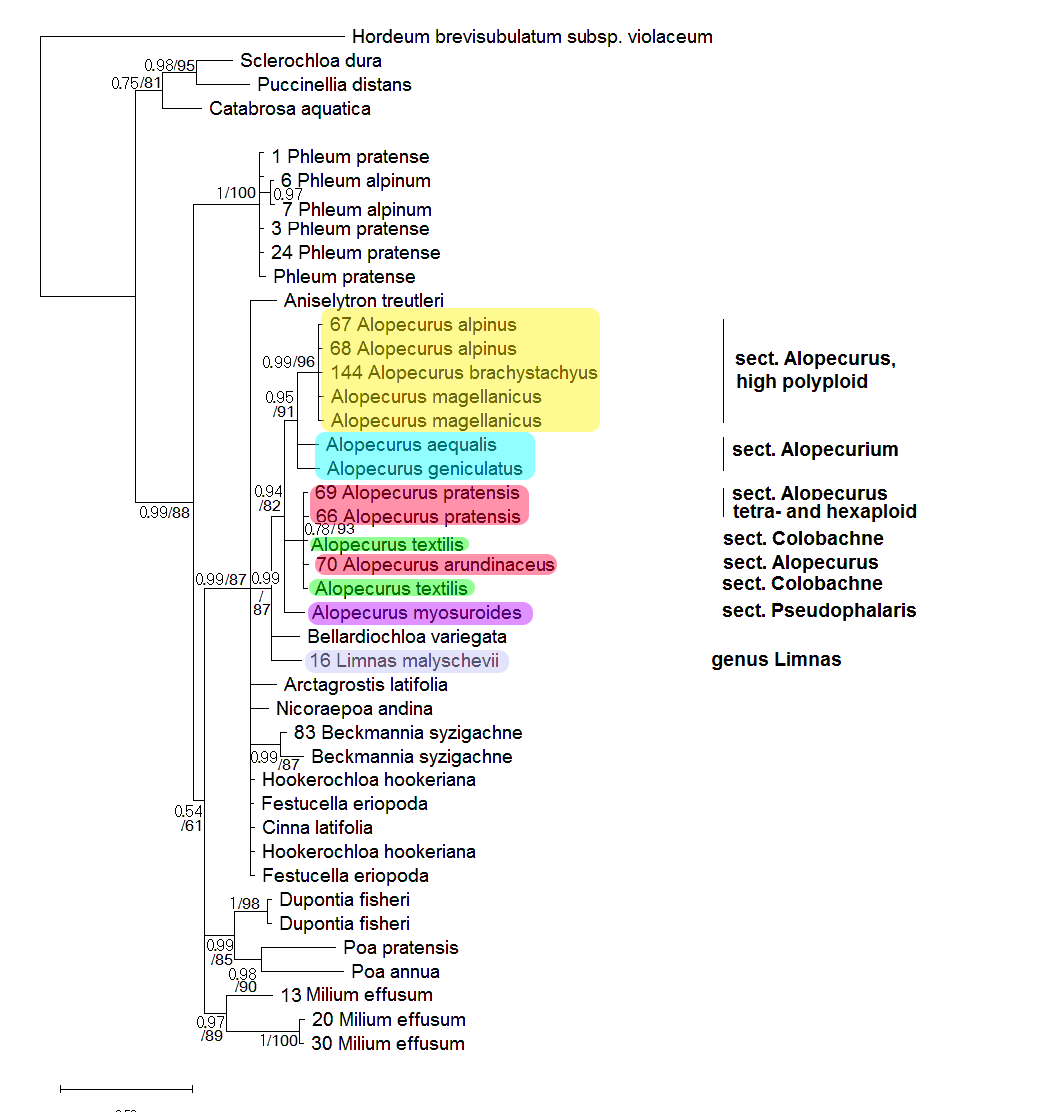

Supplement: Supplementary file 1 [file plants-13-00919-s001.zip › Alopecurus_ndhF_fi2.bmp]

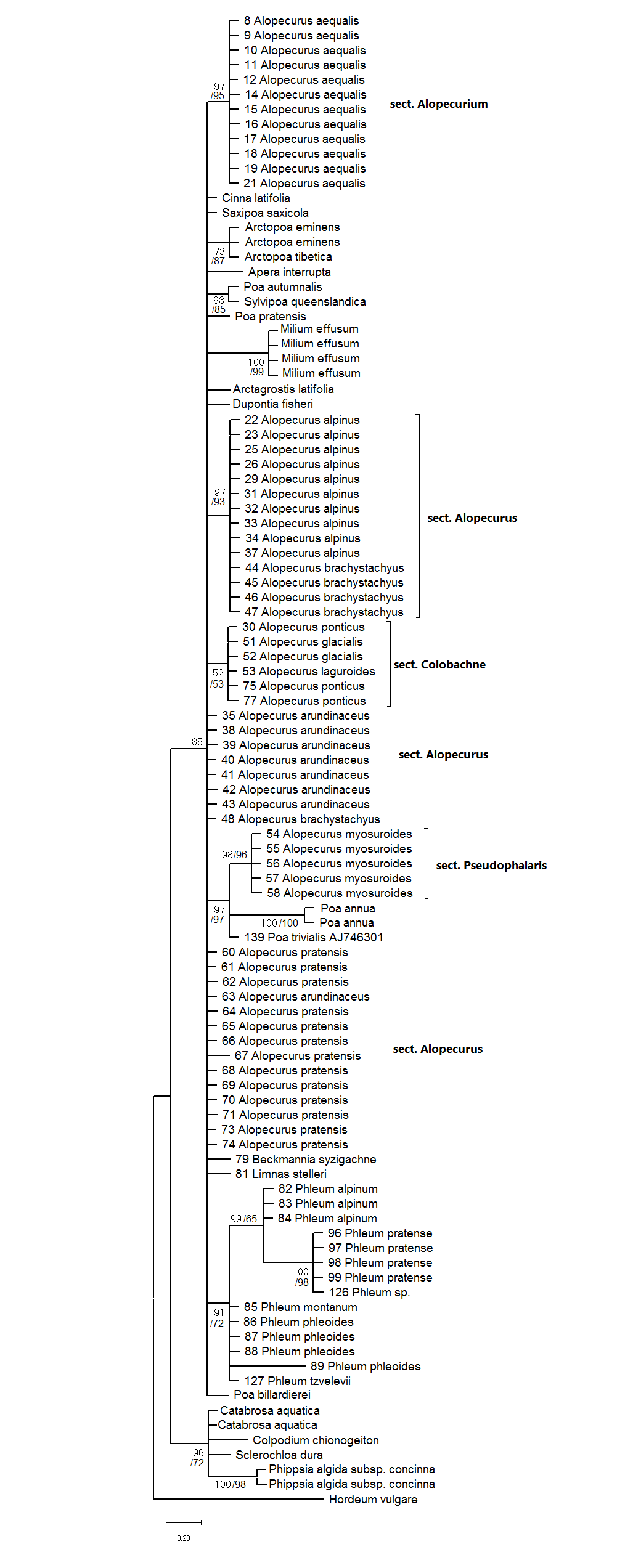

Supplement: Supplementary file 1 [file plants-13-00919-s001.zip › Alopecurus_rbcL_figure S1.png]

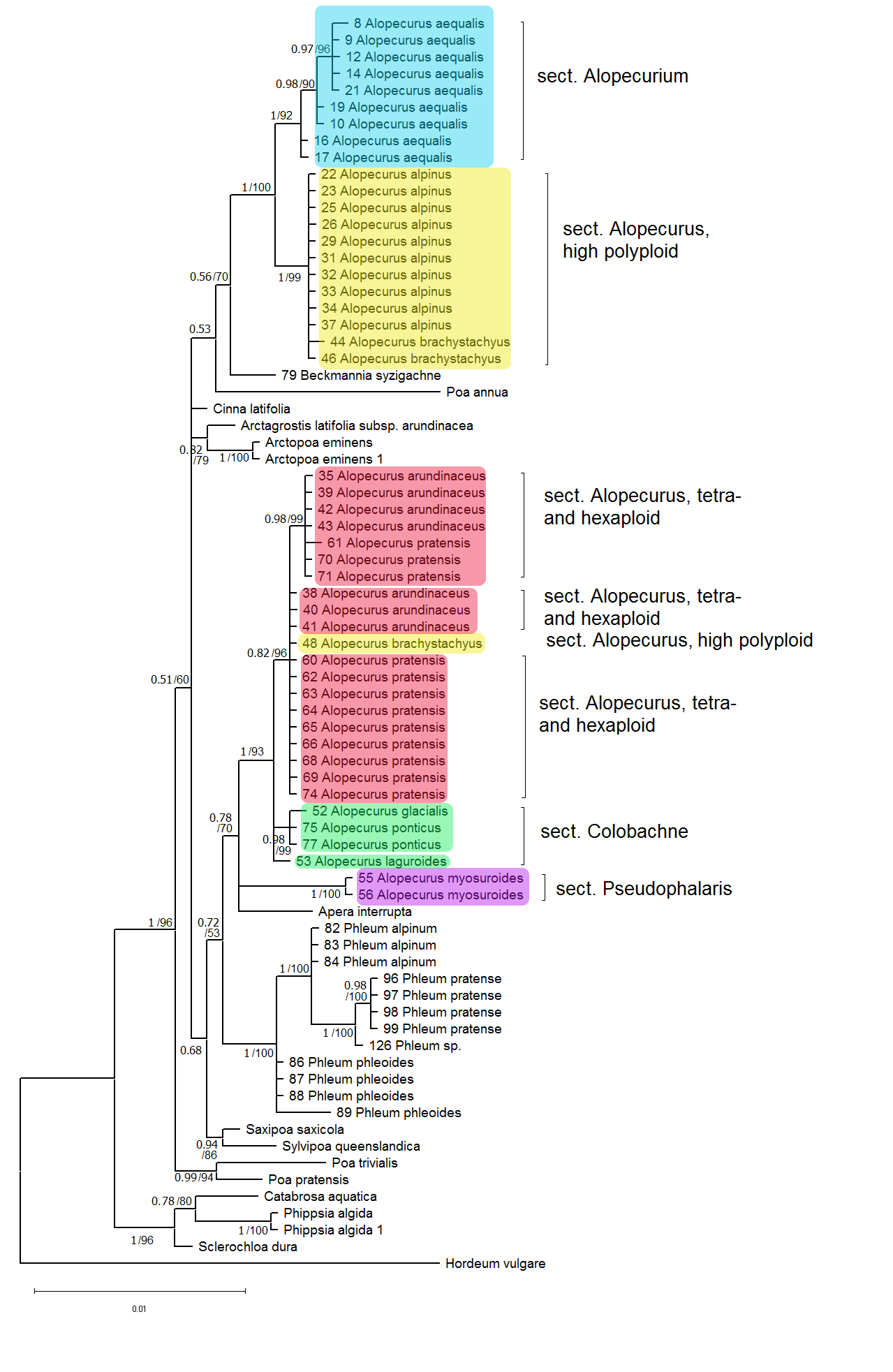

Supplement: Supplementary file 1 [file plants-13-00919-s001.zip › Alopecurus_rbcL_matK_fig1.png]

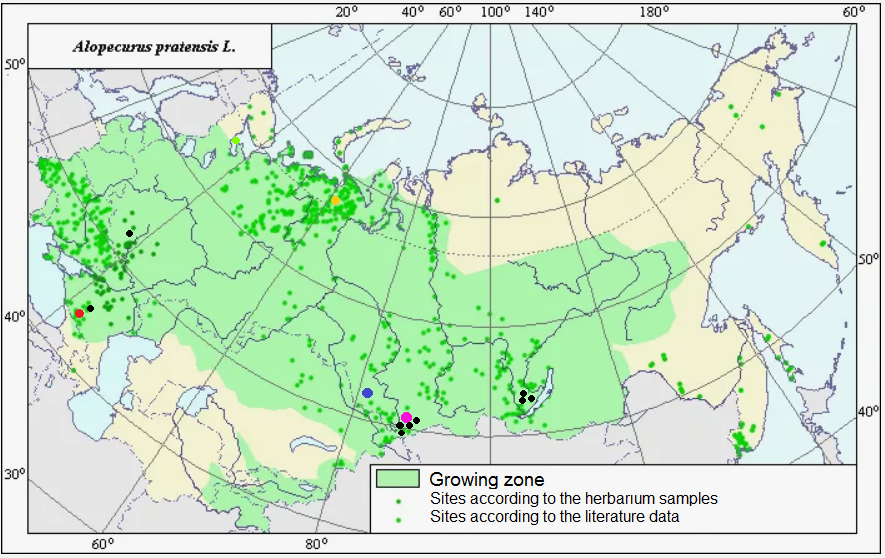

Supplement: Supplementary file 1 [file plants-13-00919-s001.zip › A_pratensis_range_with_collections_figure S8.png]

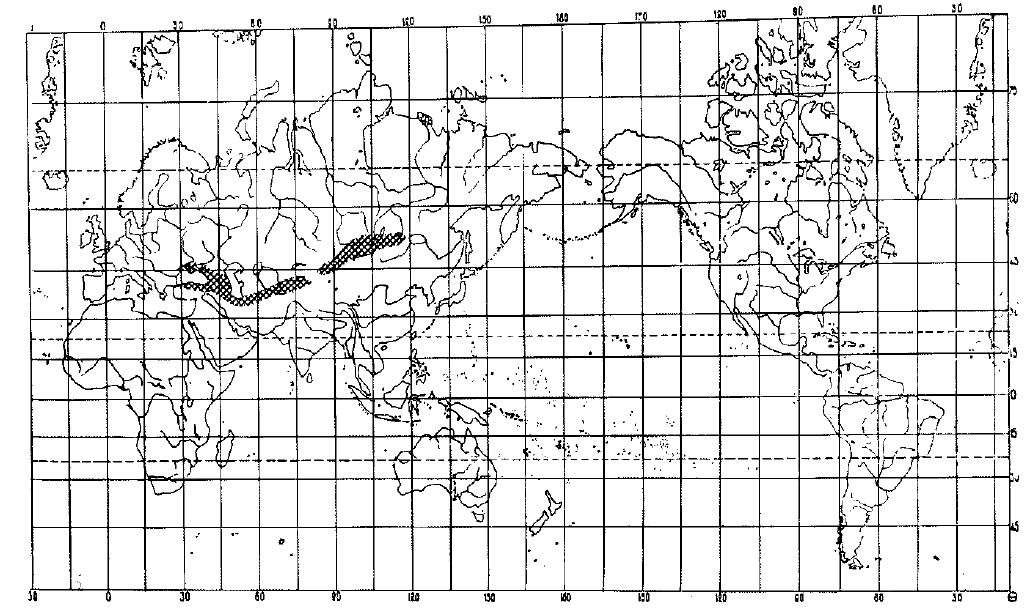

Supplement: Supplementary file 1 [file plants-13-00919-s001.zip › Colobachne_figure S9.png]

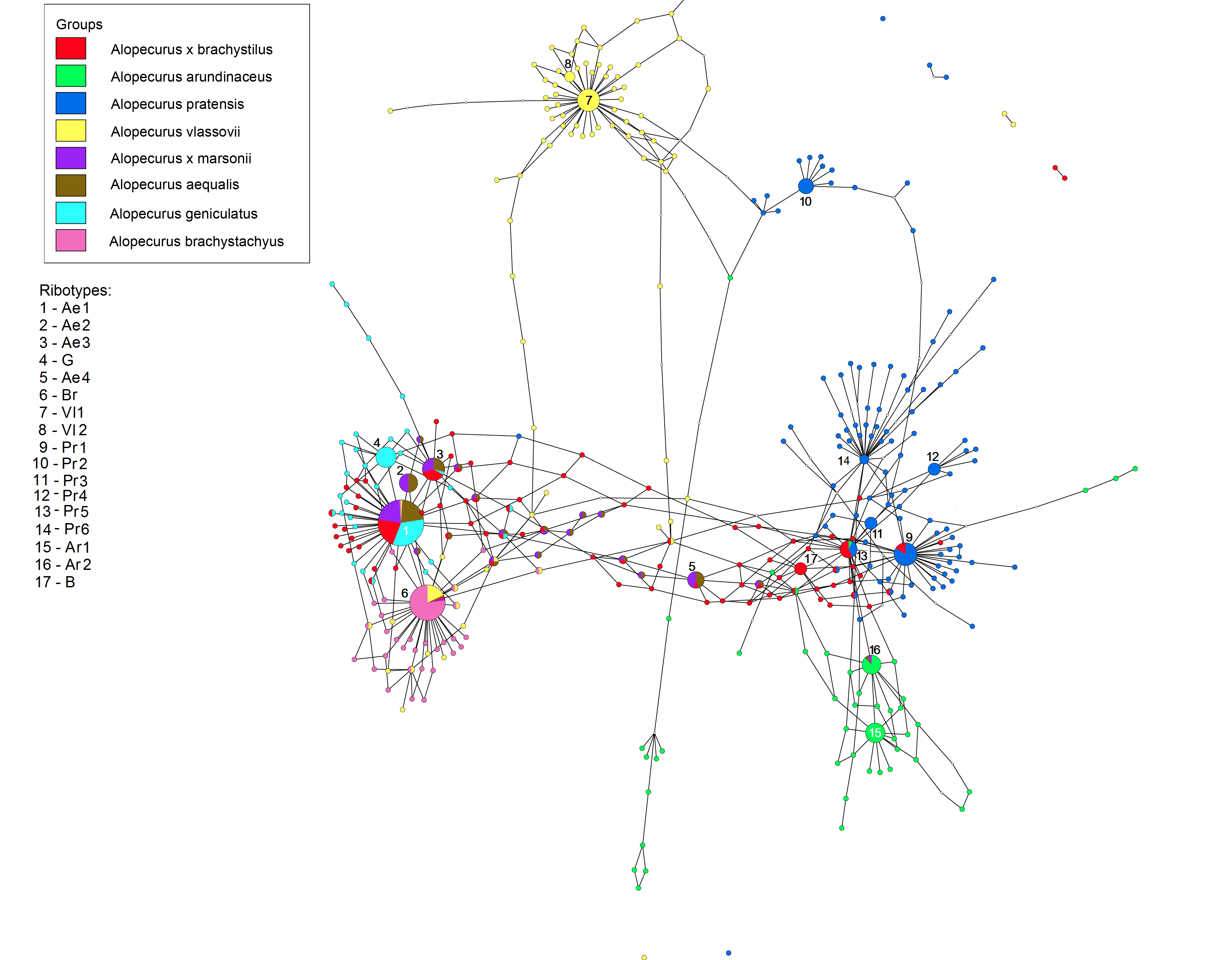

Supplement: Supplementary file 1 [file plants-13-00919-s001.zip › network_Alopecurus_figure S4_NO_NUMBERS.bmp]

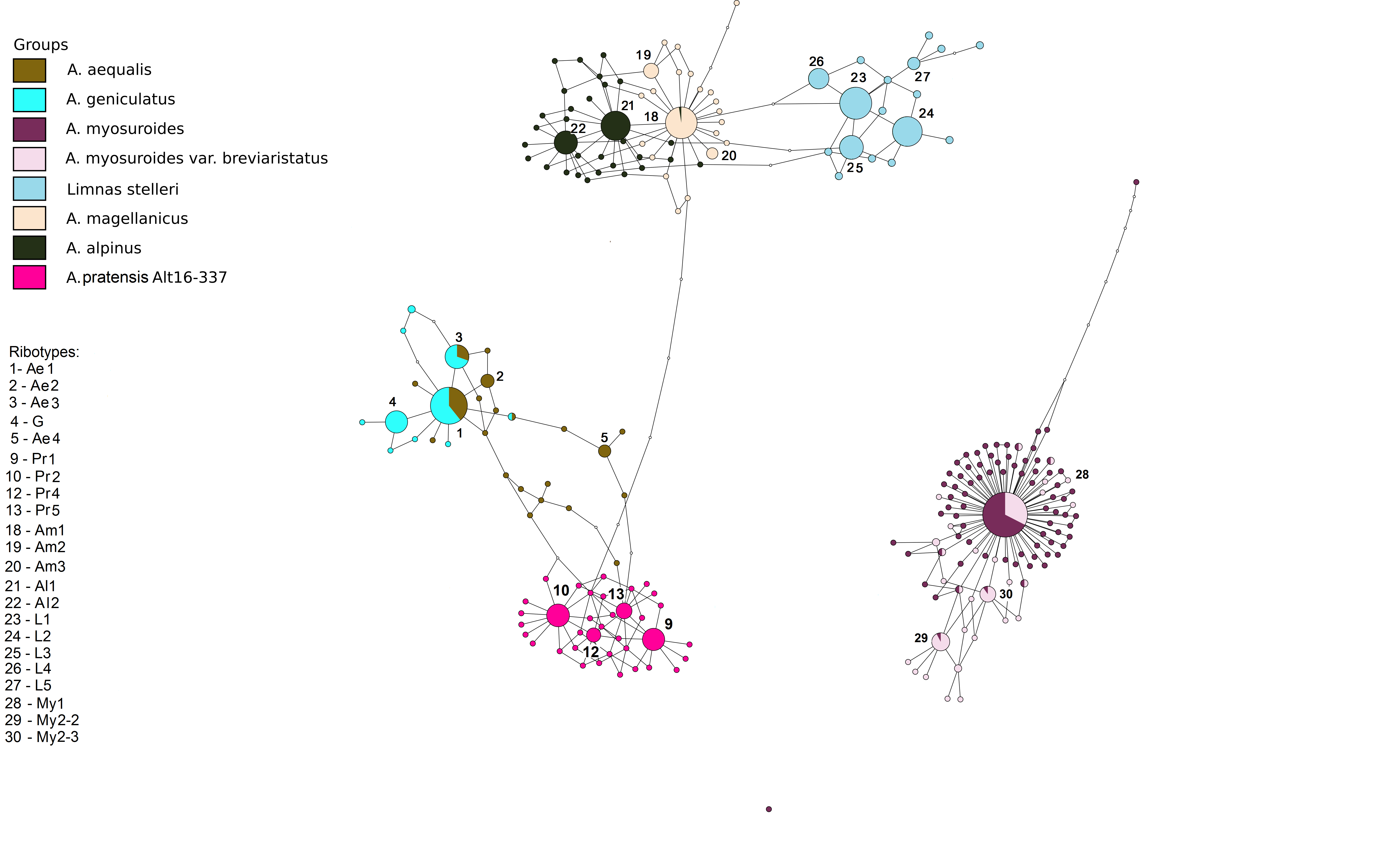

Supplement: Supplementary file 1 [file plants-13-00919-s001.zip › network_Alopecurus_figure S5_NO_NUMBERS.png]

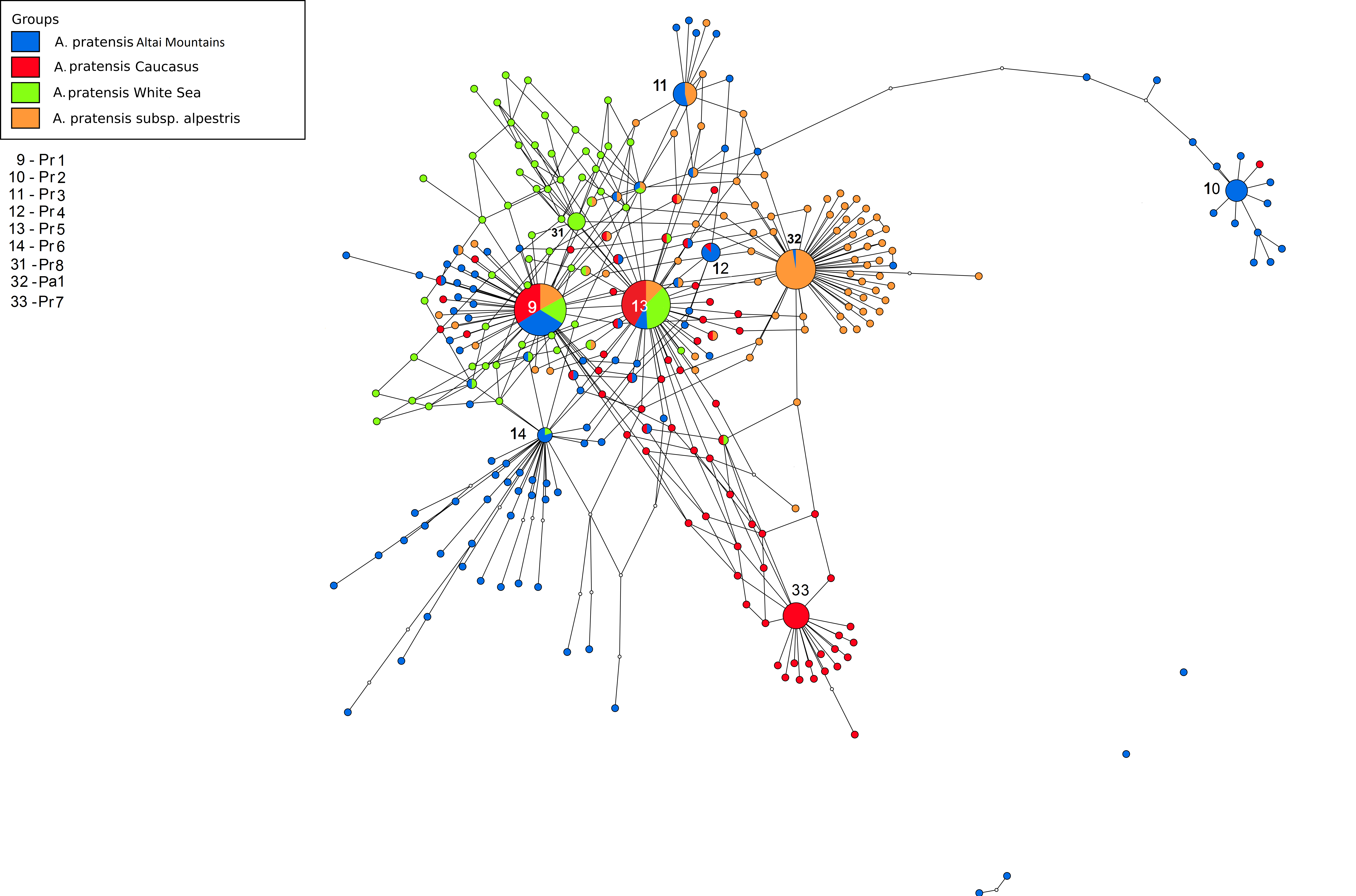

Supplement: Supplementary file 1 [file plants-13-00919-s001.zip › network_Alopecurus_figure S6_NO_NUMBERS.png]

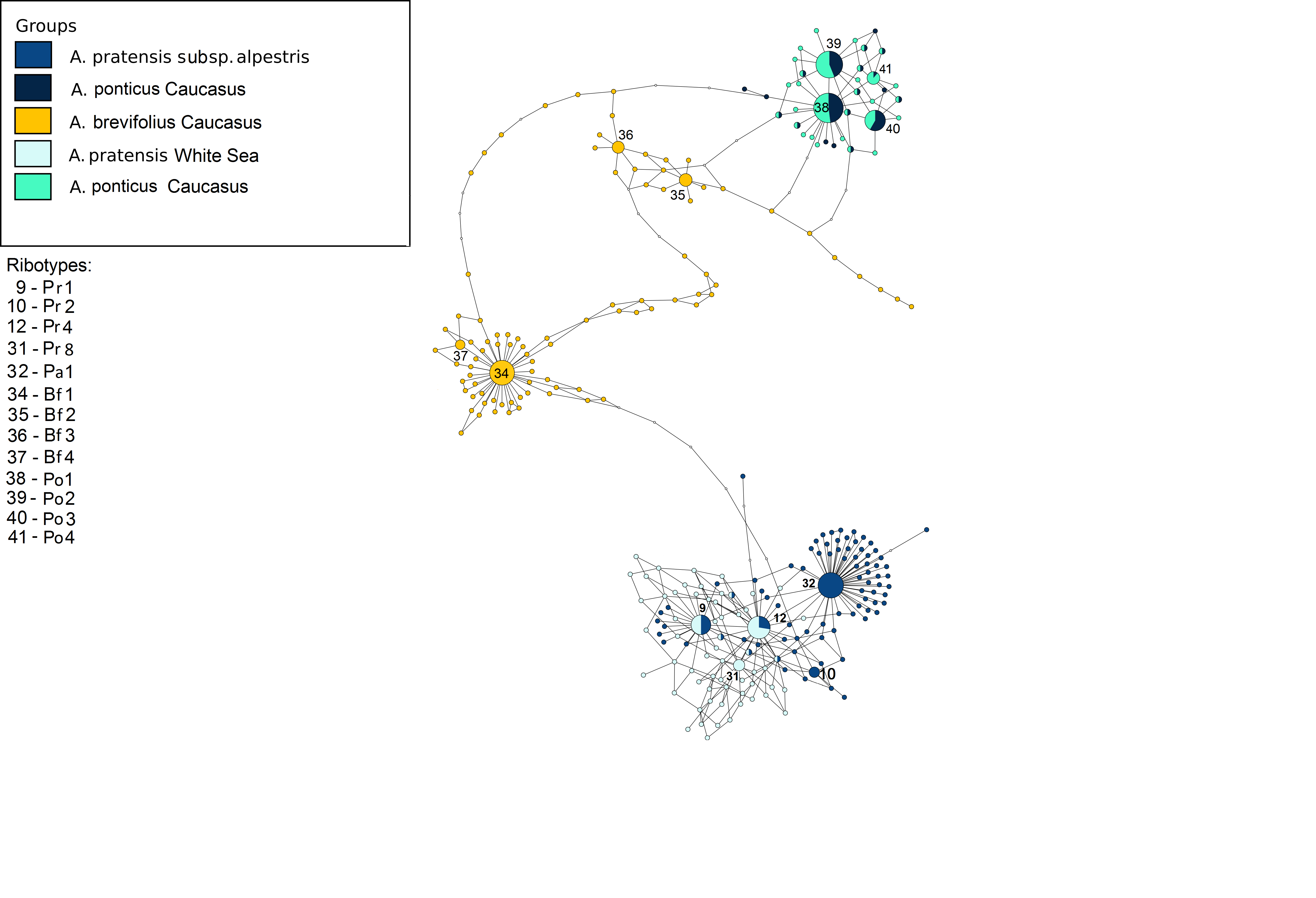

Supplement: Supplementary file 1 [file plants-13-00919-s001.zip › network_Alopecurus_figure S7_NO_NUMBERS.png]
